# Vanadium Pentoxide–Mediated Oxidation Coupled with HPLC-MS/MS for Broad-Spectrum Screening of Paralytic Shellfish Toxins in Plasma

**DOI:** 10.3390/md24100343

**Published:** 2026-09-29

**Authors:** Yangde Ma, Huilan Yu, Xiujie Liu, Bo Chen, Longhui Liang, Shilei Liu

**Affiliations:** State Key Laboratory of Chemistry for NBC Hazards Protection, Beijing 102205, China; mayangde163@163.com (Y.M.);

**Keywords:** paralytic shellfish toxins, liquid chromatography-tandem mass spectrometry, oxidation reaction, saxitoxin

## Abstract

Paralytic shellfish toxins (PSTs), potent neurotoxic alkaloids produced by marine dinoflagellates and cyanobacteria, pose severe risks to human health through contaminated seafood and water after absorption into the bloodstream. Current detection methods are limited by matrix interference or ethical concerns. Here, we report a novel screening approach using vanadium pentoxide as an oxidant to convert PSTs into characteristic oxidation products, followed by high-performance liquid chromatography-tandem mass spectrometry (HPLC–MS/MS) analysis. Under optimized conditions, vanadium pentoxide oxidizes both hydroxylated and non-hydroxylated toxins into distinct major products (P1–P7), identified by high-performance liquid chromatography–tandem high-resolution mass spectrometry (HPLC–HRMS). Vanadium pentoxide oxidation affords excellent product selectivity and, for most PSTs, superior signal intensities, enabling unambiguous differentiation of toxin subgroups. HPLC–MS/MS method in MRM mode was developed and yielded detection limits of 0.3–1.5 ng/mL for a broad spectrum of fourteen PSTs in spiked plasma, accuracy of 89.3–102.2%, and excellent repeatability with intra- and inter-day relative standard deviations (RSDs) ranging from 0.3% to 10.4%. Application of the method to samples from the First Trial OPCW (the Organisation for the Prohibition of Chemical Weapons) Biotoxin Proficiency Test successfully identified STX/neoSTX, confirming its practical utility for trace PSTs detection in complex biological matrices.

## 1. Introduction

The paralytic shellfish toxins (PSTs), derivatives of saxitoxin (STX), are a group of naturally occurring neurotoxic alkaloids primarily produced by both marine dinoflagellates and freshwater cyanobacteria, which can be classified into non-hydroxylated and hydroxylated toxins based on whether a hydroxyl group is present at the N1 position, and categorized into three generic groups based on their net charge state (Figure 1) [1,2,3,4,5]. Saxitoxin is also unique in that it is the only marine natural product listed among the controlled chemicals in the Chemical Weapons Convention [6]. Bioaccumulation of these toxins by the tissues of fish, molluscs, and crustaceans makes human consumers vulnerable to their exposure [3]. Furthermore, cyanobacterial blooms producing PSTs result in the contamination of drinking and recreational water resources [7]. The toxic effect of STX and related PSTs stems from their high affinity to voltage-gated sodium channels [8,9]. PST poisoning manifests as symptoms ranging from mild perioral paraesthesia and numbness to lethal respiratory paralysis [10]. The maximum permitted level for PSTs in bivalve molluscs is 800 μg STX equivalents per kilogram of shellfish tissue (800 μg STX eq./kg) in many countries [11].

Various detection techniques have been used to monitor commercially harvested shellfish and manage hazards associated with PSTs. Initially, the mouse bioassay (MBA) method was developed for detecting paralyzing toxins [12,13,14,15]. However, the limitations, such as inability to distinguish toxin analogs, low selectivity, and animal ethics controversies, restrict their widespread application. Subsequently, enzyme-linked immunosorbent assay (ELISA) method [16,17,18], thin-layer chromatography [19], capillary electrophoresis [20], and high-performance liquid chromatography-fluorescence detection (HPLC–FLD) [4,21,22,23,24,25,26] have been proposed for the determination of PSTs. Among them, the HPLC–FLD method (AOAC 2005.06) has become the official European Union reference method for the determination of PSTs in shellfish. Hydrogen peroxide and periodate are used in the HPLC–FLD method (AOAC 2005.06) to oxidize PSTs into fluorescent derivatives [23], which exhibit better retention on reversed-phase liquid chromatographic columns (RPLC) than native PSTs.

Currently, high-performance liquid chromatography–tandem mass spectrometry (HPLC–MS/MS) has emerged as the dominant analytical approach in the detection of PSTs. Compared with HPLC–FLD, which solely relies on retention time for identification and suffers from fluorescence interferences originating from sample matrices, HPLC-MS/MS offers superior accuracy and specificity by using both retention times and characteristic fragment ions for confirmation, even in the absence of reference chemicals. Direct detection of PSTs by hydrophilic interaction liquid chromatography–tandem mass spectrometry (HILIC–MS/MS) has been widely used [27,28,29,30,31], although it is susceptible to matrix interferences. Currently, the oxidized products of some PSTs have been identified by HPLC-MS/MS [32,33], which makes it possible to screen PSTs by detecting these oxidized products using HPLC-MS/MS.

Upon entering the human body, PSTs rapidly distribute to various organs via the bloodstream and trigger diverse clinical manifestations, such as dyspnea, ataxia, dystonia, paresthesia, tachycardia, hypotension, and asthenia. Among these symptoms, dysphagia and dysarthria are regarded as characteristic clinical features. Accordingly, timely and accurate identification of PSTs in biological fluids such as plasma is critical for diagnosis, symptom management, and the delivery of appropriate supportive treatment for patients.

In this study, vanadium pentoxide was used as the oxidizing reagent to convert PSTs in plasma to characteristic oxidation products. These products were structurally characterized by high-performance liquid chromatography–tandem high-resolution mass spectrometry (HPLC-HRMS), and the HPLC-MS/MS method in MRM mode based on these typical products was established for targeted screening of PSTs in plasma.

## 2. Results and Discussion

### 2.1. Identification of Vanadium Pentoxide Oxidation Products of PSTs

#### 2.1.1. Oxidation of STXs

Here, we performed oxidation of STX and dcSTX with vanadium pentoxide in basic solution (Figure S1A,B), and the reaction products were subsequently characterized using HPLC-HRMS. The chromatograms and MS/HRMS mass spectra of STX oxidation products are shown in Figure 2A and Figure S2A, respectively. The oxidation of STX produced the minor product P1 and major product P2 [34]. Meanwhile, dcSTX was extensively oxidized into P1 (Figure 2B and Figure S2B). These results indicate that vanadium pentoxide has the potential to serve as an oxidant for STXs (Figure 1). We further applied vanadium pentoxide to oxidize other STXs, such as neoSTX and dcneoSTX. As shown in Figure S1C and Figure 2C, neoSTX mainly yielded product P2, accompanied by small quantities of P1 and P3 (the corresponding mass spectra are shown in Figure S2C). The dcneoSTX was also oxidized into major product P1 and minor product P3 (Figure 2D, Figures S1D and S2D). The results demonstrated that during the vanadium pentoxide oxidation reaction, P2 represents the predominant oxidation product of STX and neoSTX, whereas P1 constitutes the main oxidation product of dcSTX and dcneoSTX. These products were formed by cleavage of the C4–C12 bond in the parent compounds, accompanied by the loss of four hydrogen atoms and the elimination of the N-1-hydroxy group (if present) [32,34].

#### 2.1.2. Oxidation of GTXs

Based on the above results, we hypothesized that vanadium pentoxide serves as an excellent alternative to periodic acid for the oxidative detection of hydroxylated and non-hydroxylated toxins. Accordingly, this approach was applied to GTXs (Figure 1), and the products were characterized by HPLC-HRMS.

GTX1 and GTX4 are epimeric compounds. Since pure GTX1 and GTX4 references could not be obtained, an oxidation reaction was performed using a GTX1/4 mixed reference. After vanadium pentoxide oxidation (Figure S1E,F) [35], a predominant product designated as P4 with a molecular ion at *m*/*z* 312.1052, and a minor product designated as P5 with a molecular ion at *m*/*z* 269.0993 in the full-scan mass spectra. The chromatograms and MS/HRMS spectra of GTX1/4 oxidation products are shown in Figure 2E and Figure S2E, respectively. P4 exhibited a response intensity nearly two orders of magnitude higher than that of P5. The proposed fragmentation pathways are shown in Figure S2E based on the fragmentation ions obtained. Notably, products P5 and P3 share the same molecular weight, but obvious differences were observed in their MS/HRMS fragments (Figure S2C,E), which further verify them as distinct compounds. Since GTX1/4 contains sulfonate groups, we also detected the oxidation product in negative ion mode, and a peak at *m*/*z* 390.0474 was observed at the retention position of P4, and the corresponding MS/HRMS spectrum is shown in Figure S2E. By integrating mass spectrometric data acquired under both positive ion and negative ion modes, the structure of P4 was further corroborated. A characteristic neutral loss of SO_3_ (Δm = 80 Da) from the sulfonate group is clearly observed in positive ion mode [36,37]. The product P4 was formed by cleavage of the C4–C12 bond in the parent compound, accompanied by the loss of four hydrogen atoms and the elimination of the N-1-hydroxy group. It should be noted that P4 generated from GTX1 and GTX4, respectively, are also epimers. Multiple attempts failed to achieve their chromatographic separation, and thus they were regarded as one single product in this study. This treatment is also applicable to products P5 and P7 in the following sections.

The oxidation product distribution of GTX2/3 was consistent with that of GTX1/4 (Figure S1G,H). The chromatogram is shown in Figure 2F, and the corresponding MS/HRMS mass spectra are displayed in Figure S2F. The predominant product P4 was formed by cleavage of the C4-C12 bond in the parent compound, accompanied by the loss of four hydrogen atoms. Product P4 represents the common major characteristic oxidation product of GTX1/4 and GTX2/3, and could potentially act as a marker to confirm the presence of these toxins.

After vanadium pentoxide oxidation, GTX5 was oxidized to products P1 and P6 (Figure S1I) with molecular ions at *m*/*z* 253.1043 and 376.0675, respectively. Their intensities were comparable. The chromatogram and MS/HRMS mass spectra of GTX5 oxidation products are shown in Figure 2G and Figure S2G, respectively. The proposed fragmentation pathway is shown in Figure S2G based on the fragmentation ions obtained. Product P6 was formed from the parent toxin via cleavage of the C4-C12 bond together with the loss of four hydrogen atoms. Especially, product P6 was also detected in negative ion mode; the corresponding mass spectra are shown in Figure S2G. The oxidation product distribution of GTX6 was consistent with that of GTX5 (Figure S1J). The chromatogram is shown in Figure 2H, and the corresponding MS/HRMS mass spectra are displayed in Figure S2H. Product P6 represents the characteristic oxidation product of GTX5 and GTX6, and could potentially act as a marker for verifying these toxins.

Upon oxidation of dcGTX2/3 (Figure S1K,L), product P5 with a molecular ion at *m*/*z* 269.0993 was obtained. The chromatogram and MS/HRMS mass spectra of dcGTX2/3 oxidation products are shown in Figure 2I and Figure S2I, respectively. In the negative ion mode, product P5 displayed a molecular ion peak at *m*/*z* 347.0415. The proposed fragmentation pathway is shown in Figure S2I based on the fragmentation ions obtained. Owing to the presence of a sulfonate group, facile neutral loss of SO_3_ (Δm = 80 Da) is observed in positive ion ESI mode [35,36]. Product P5 was formed by cleavage of the C4-C12 bond in the parent compound, accompanied by the loss of four hydrogen atoms.

#### 2.1.3. Oxidation of C1/2 Toxins

As shown in Figure 2J and Figure S2J, the oxidation of the C1/2 toxins yielded products P5 and P7 (Figure S1M,N) with molecular ions at *m*/*z* 269.0993 and *m*/*z* 392.0619, respectively. Their responses are comparable. In the negative ion mode, products P5 and P7 displayed ion peaks at *m*/*z* 347.0415 and *m*/*z* 470.0042, respectively. The proposed fragmentation pathway is shown in Figure S2J. Owing to the presence of a sulfonate group, facile neutral loss of SO_3_ (Δm = 80 Da) is observed in positive ion ESI mode [36,37]. The product P7 was formed by cleavage of the C4–C12 bond in the parent compound, accompanied by the loss of sulfonate groups at C11. It represents the characteristic oxidation product of C1 and C2, and could potentially act as a marker for verifying these toxins.

Collectively, these experimental results demonstrate that multiple toxin analogs share common oxidation products, with the majority affording one major product. Accordingly, detection of these characteristic major products can be utilized for qualitative and quantitative analysis of target toxins.

### 2.2. Reaction Condition Optimization

Subsequently, an HPLC-MS/MS method in MRM mode was developed based on the results of HPLC-HRMS. The collision energy values and ion transitions are shown in Table S1. The precursor-product ion transition with the highest signal was selected for quantification, while the secondary product ion and the abundance ratio of the two transitions were used for confirmation.

#### 2.2.1. The pH of the Reaction Buffer

With the HPLC-MS/MS (MRM) method in hand, we investigated the influence of the pH of the reaction buffer, reaction temperature, and time on the reaction products [38]. We carried out reaction optimization within the pH range of 9.6–12.2 on fourteen kinds of PSTs, and the results are shown in Figure S3. It can be seen that the optimum pH for the hydroxylated toxins (neoSTX, dcneoSTX, GTX1/4, GTX6) is about 10.2, and the optimum pH for the non-hydroxylated toxins (STX, dcSTX, GTX2/3, dcGTX2/3, GTX5, C1/2) is about 11.6. We hope that there is a pH at which it would produce a single product for GTX5, GTX6, and C1/2 predominantly. However, there were no apparent variations in product distribution across all tested pH conditions. The optimum pH for the non-hydroxylated toxins is higher than that for the hydroxylated toxins. This trend is consistent with that reported for the periodate oxidation reaction [38]. The difference is that the optimum pH of the periodate reaction is lower than that of the vanadium pentoxide-mediated reaction. The reason may be that here we optimize the pH of the reaction buffer, while vanadium pentoxide can further interact with alkaline buffer solutions at high temperature, thereby altering the final pH of the reaction solution. Nevertheless, during toxin screening of actual samples, the specific toxin components are unknown, so the most suitable pH condition cannot be predetermined. A universal pH is required for the oxidation reaction in PSTs screening. For instance, a buffer at pH 8.2 is routinely used for periodate oxidation [23]. Here, sodium hydrogen phosphate buffer (0.3 M, pH 11.6) was adopted for the screening reaction.

#### 2.2.2. Reaction Temperature and Time

Temperature exerted a substantial effect on the oxidation reaction. STX, GTX1/4, and GTX5 were selected as model toxins for the optimization of reaction temperature and time. The corresponding results are presented in Figure 3. The MS response intensities of the major product measured by HPLC-MS/MS after 60 min of reaction at different temperatures are shown in Figure 3A. The response of the major product increased significantly with increasing temperature. Comparable responses were obtained at 80 °C and 90 °C, so the reaction temperature was set to 80 °C. On this basis, reaction time was further optimized, and the results are illustrated in Figure 3B. No obvious further enhancement in the response of the major product was observed when the reaction time was prolonged beyond 15 min. Accordingly, 15 min was adopted as the optimal reaction time.

#### 2.2.3. Reaction Products and Distribution

Fourteen PSTs were subjected to oxidation by vanadium pentoxide and periodate (AOAC 2005.06) [23]. The oxidation products were determined by HPLC-MS/MS, and the corresponding results are displayed in Figure S4. The final concentrations of the corresponding PSTs were identical in both oxidation reactions. The peak areas of the main oxidation products are shown beside each peak. For STX and neoSTX (Figure S4A,B), P2 was the predominant product in both vanadium pentoxide and periodate oxidation. The area of peak P2 in vanadium pentoxide oxidation was approximately 1.8–5.5 times higher than that in the periodate oxidation. In addition, periodate oxidizes neoSTX to yield P1 and P3 with considerable signal responses, leading to poor selectivity. For dcSTX and dcneoSTX (Figure S4C,D), P1 was the predominant product in both oxidation reactions. The peak area of P1 in vanadium pentoxide oxidation was approximately 2.5–90 times higher than that in the periodate oxidation.

P4 was the unique product formed upon oxidation of GTX1/4 and GTX2/3 (Figure S4E,F). Upon oxidation of GTX1/4, vanadium pentoxide oxidation afforded product P4 with an approximately 4-fold higher peak area than that obtained via periodate oxidation. In addition, vanadium pentoxide oxidation of GTX1/4 exhibited excellent selectivity, yielding predominantly P4 with negligible amounts of P5. In comparison, periodate oxidized GTX1/4 to yield some P5. Exceptionally, both oxidants could convert GTX2/3 to P4, but the area of peak P4 obtained under vanadium pentoxide oxidation is half of that observed from periodate oxidation. Meanwhile, P5 was the predominant oxidation product of dcGTX2/3 for both reactions (Figure S4G), and its intensity in vanadium pentoxide oxidation was roughly half that of the periodate oxidation. Although vanadium pentoxide oxidation of GTX2/3 and dcGTX2/3 yielded lower abundances of their main products relative to periodate, it exhibited better product selectivity in oxidizing GTXs. These findings reveal that vanadium pentoxide oxidation enables the discrimination of GTX1/2/3/4 from other PSTs.

P6 was the characteristic product from GTX5 and GTX6 oxidation. Upon oxidation of GTX5 (Figure S4H), both oxidation reactions yield P1 and P6, and their abundances from vanadium pentoxide oxidation are nearly 3 times those from periodate oxidation. Notably, vanadium pentoxide oxidation of GTX6 afforded P1 and P6 (Figure S4I), as observed for GTX5, while periodate oxidation of GTX6 afforded P1 and P2, rather than P1 and P6. These findings reveal that vanadium pentoxide oxidation enables the discrimination of GTX5/6 from other PSTs, whereas periodate oxidation cannot.

P7 was the characteristic product from C1/2 oxidation (Figure S4J). Oxidation of C1/2 gave identical product profiles for both reactions, with P4 and P7 formed under both conditions. The product intensities from periodate oxidation are about 3 times those obtained in vanadium pentoxide oxidation.

Collectively, vanadium pentoxide oxidation delivers more main products for most PSTs, whereas periodate favors more reaction products only for GTX2/3, dcGTX2/3, and C1/2. Most notably, vanadium pentoxide oxidation of PSTs achieves better product selectivity and specificity, which is well suited for the screening of PSTs. The main oxidation products and the percentage of each peak are shown in Table S2. The P2, P4, P6, and P7 represent the characteristic oxidation products of STX/neoSTX, GTX1/2/3/4, GTX5/6, and C1/2. Both GTX5 and GTX6 produce two oxidation products, P1 and P6, with the same relative peak area ratio of 0.526 (P1/P6). C1/2 toxins produce two oxidation products, P5 and P7, with the relative peak area ratio of 0.972 (P5/P7). The presence of P5 and P7 and their relative peak area ratio could be used to determine whether dcGTX2/3 and/or C1/2 are present in the sample. This method can be applied in screening dcSTX/dcneoSTX and GTX5/6.

For example, Mix1, a mixture of STX, dcSTX, GTX1/4, GTX5, and dcGTX2/3, was oxidized by vanadium pentoxide, and the HPLC-MS/MS analysis results are shown in Figure S4K. The appearance of peaks P2, P4, and P6 indicates that the sample contains STX/neoSTX, GTX1/2/3/4, and GTX5/6, respectively. The presence of peak P5 and absence of peak P7 indicates the existence of dcGTX2/3 and the absence of C1/2. The simultaneous presence of peaks P1 and P6 confirms the existence of GTX5/6. dcSTX/dcneoSTX may also be present. The area of peak P1 originating from dcSTX/dcneoSTX oxidation can be determined by Equation (1). Here, 0.526 represents the peak area ratio of P1 to P6 generated by the oxidation of GTX5/6.

Area(P1_dcSTX/dcneoSTX_) = Area(P1) − Area(P6) × 0.526, (1)

Mix2, a mixture of STX, neoSTX, dcSTX, dcneoSTX, GTX1/4, GTX2/3, GTX5, GTX6, dcGTX2/3 and C1/2, was oxidized by vanadium pentoxide, and the HPLC-MS/MS analysis results are shown in Figure S4K. The appearance of peaks P2, P4, P6, and P7 indicates that the sample contains STX/neoSTX, GTX1/2/3/4, and GTX5/6, respectively. The simultaneous presence of peaks P1 and P6 confirms the existence of GTX5/6, and dcSTX/dcneoSTX may also exist. The area of peak P1 originating from dcSTX/dcneoSTX oxidation can be determined by Equation (1). In addition, the simultaneous presence of peaks P5 and P7 indicates that C1/2 and dcGTX2/3 may also exist. The area of peak P5 originating from dcGTX2/3 oxidation can be determined by Equation (2). Here, 0.972 represents the peak area ratio of P5 to P7 generated by the oxidation of C1/2.

Area(P5_dcGTX2/3_) = Area(P5) − Area(P7) × 0.972, (2)

For toxicity estimation, each oxidation product peak was attributed to the toxin with the maximum TEF as a conservative estimate. For example, we can conservatively assume that peak P4 originates entirely from the oxidation of GTX1, as GTX1 has the highest TEF among GTX1/2/3/4 and their oxidation efficiencies are close. The total PST toxicity is calculated as the sum of each toxin concentration multiplied by its TEF [21]. This calculated value is only suitable for determining whether the sample is below the regulatory limit and does not represent an accurate toxicity value.

### 2.3. Method Validation

The performance of the proposed method was established by employing assays with blank plasma and spiked plasma samples, and the results are shown in Table 1. The whole procedure of the developed method includes solid-phase extraction cleanup, vanadium pentoxide oxidation, and HPLC-MS/MS (MRM) analysis. The results showed that no impurity peaks corresponding to any of the target compounds were detected in plasma blanks, demonstrating the good specificity of this method.

#### 2.3.1. Linearity, LODs and LOQs

Matrix-matched PST calibration curves were prepared in pre-processed blank plasma using serial reference concentrations of PSTs against the response intensities of their major oxidation products formed upon vanadium pentoxide oxidation. The linear ranges of the calibration curves ranged from LOQ to 50 ng/mL or 75 ng/mL. All correlation coefficients were greater than 0.9958. The limits of detection (LOD) for the fourteen PSTs ranged from 0.3 to 1.5 ng/mL in human plasma, and limits of quantitation (LOQ) for the fourteen PSTs ranged from 1 to 5 ng/mL in human plasma.

#### 2.3.2. Accuracy and Precision

The intra-day and inter-day precisions were obtained by spiking the human plasma with two levels (LOQ and 50 ng/mL) of PSTs, performing detection for four consecutive days and six replicates per day, respectively. Accuracy was calculated in terms of recoveries (R%). As shown in Table 1, the intra-day accuracy at LOQ was in the range of 91.0% to 100.6% with RSD in the range of 0.4% to 7.9%, and the intra-day accuracy at 50 ng/mL was in the range of 94.5% to 102.2% with RSD in the range of 0.6% to 9.8%. The inter-day accuracy at LOQ was in the range of 94.9% to 103.4% with RSD in the range of 2.0% to 10.4%, and the inter-day accuracy at 50 ng/mL was in the range of 89.3% to 101.4% with RSD in the range of 0.3% to 9.3%. The results manifested that this method had good precision and was well suited for analysis of trace PSTs in plasma.

#### 2.3.3. Stability

The stability of the major oxidation product was assessed by storing the reference solutions (LOQs and 50 ng/mL) after vanadium pentoxide oxidation at 15 °C for 48 h, and comparing the response intensities before and after incubation. As shown in Table S3, the stabilities were greater than 84.7% for all main products of PSTs.

#### 2.3.4. Application

In addition, this method was applied in screening the plasma samples from the First Trial OPCW (the Organisation for the Prohibition of Chemical Weapons) Biotoxin Proficiency Test. The results of the HPLC-MS/MS analysis are presented in Figure 4. Only product P2 was detected in samples TBT011, TBT013, TBT014, and TBT015, suggesting that STX and/or neoSTX are present in these samples. The OPCW-issued spiking list of the First Trial OPCW Biotoxin Proficiency Test showed that samples TBT011, TBT013, TBT014, and TBT015 all contain STX, validating the practicality of the method.

#### 2.3.5. Limitations of the Method Studied

To date, numerous methods have been employed for PST detection in shellfish [21,22,23,24,25,26]. However, only HILIC-MS/MS approaches have been reported for the detection of PST in plasma [39]. As listed in Table S4, the present method exhibits lower LODs in plasma than HILIC-MS/MS approaches, which makes it suitable for the screening of low-abundance PSTs in plasma. HILIC-MS/MS is advantageous in that it offers higher detection efficiency and enables simultaneous analysis of multiple toxins without solid-phase extraction cleanup and derivatization. However, this method requires multiple reference standards to construct numerous calibration curves, which imposes a heavy workload. Therefore, it is often employed for accurate quantification and for calculating total toxicity equivalents based on TEFs. For pre-column oxidation approaches, multiple solid-phase extraction cleanups together with peroxide oxidation and periodate oxidation can solve the issue of toxin overlap, yet the overall analytical workflow is complicated. These methods rely solely on retention time for identification and suffer from fluorescence interferences originating from sample matrices. Although post-column oxidation methods could detect trace-level PSTs and avoid the overlap of oxidation products, it requires dedicated post-column reaction equipment, careful optimization of derivatization conditions, and fresh preparation of reagents. In addition, it has limited capability for structural confirmation and may suffer from matrix interference. Although the present method cannot identify individual PSTs, it is capable of determining their toxin subgroup. Therefore, it is well suited for screening PSTs in plasma rather than precise quantification. Nevertheless, the maximum toxicity equivalents can be estimated based on TEFs.

## 3. Materials and Methods

### 3.1. Materials

LC-MS grade acetonitrile (ACN), methanol (MeOH), water (H_2_O), formic acid (FA), ammonium formate (AF), and ammonium bicarbonate (NH_4_HCO_3_) were supplied by Merck (Darmstadt, Germany). Acetic acid (CH_3_COOH, AcOH), human plasma, potassium dihydrogen phosphate, and dipotassium hydrogen phosphate were purchased from Sigma-Aldrich (Saint Louis, MO, USA). Ammonium hydroxide (NH_3_·H_2_O, analytical reagent, 25%) and periodic acid were obtained from J&K Scientific Ltd. (Beijing, China). Supelclean^TM^ ENVI-Carb^TM^ (3 cc, 250 mg) was purchased from Supelco Analytical (Bellefonte, PA, USA). The Certified reference chemicals (Table 1) including STX (61.4 μM), neoSTX (65.1 μM), dcSTX (65.3 μM), dcneoSTX (30.4 μM), GTX1/4 (62.3 and 14.7 μM, respectively), GTX2/3 (102.6 and 43.5 μM, respectively), GTX5 (53.6 μM), GTX6 (13.2 μM), dcGTX2/3 (107 and 21 μM, respectively) and C1/2 (93.8 and 27.4 μM, respectively) were acquired from the Canadian National Research Council (Halifax, NS, Canada).

### 3.2. Plasma Sample Preparation

A plasma sample (200 μL) in the sealed polypropylene tube was adjusted to about pH 11 with NH_3_·H_2_O. A 250 mg ENVI-Carb^TM^ cartridge (Supelco, Bellefonte, PA, USA) was used for sample purification according to the reported method. The plasma sample was loaded onto the ENVI-Carb^TM^ cartridge preconditioned with 2.0 mL of 20%ACN containing 1% AcOH and 2.0 mL of 50 mM NH_4_HCO_3_. The cartridge was washed with 2.0 mL of 50 mM NH_4_HCO_3_ to remove the background interference. The analyte was eluted with 2.0 mL of 20%ACN containing 1% AcOH. The eluent was collected and evaporated to dryness at 30 °C using a centrifugal vacuum evaporator. Finally, the sample was reconstituted with 50 μL water.

### 3.3. Vanadium Pentoxide Oxidation

An aliquot of 10 μL sample in a 1.5 mL Eppendorf tube was mixed with 2 μL of 0.3 M ammonium formate aqueous solution, 30 μL of potassium phosphate aqueous solution (0.2 M, pH 11.6), and 20 μL of aqueous vanadium pentoxide suspension (5 mg/mL). After incubation at 80 °C with shaking at 1500 rpm for 15 min, the resulting solution was cooled to room temperature and centrifuged at 12,000 r/min for 5 min. The supernatant was collected and transferred to a microvial for HPLC-MS analysis. The pH of aqueous potassium phosphate solution, reaction temperature, and reaction time were altered when optimizing the reaction conditions.

### 3.4. Periodate Oxidation

Equal volumes of 0.03 M periodic acid, 0.3 M ammonium formate solution, and 0.3 M potassium phosphate were mixed in a 1.5 mL Eppendorf tube, and the pH was adjusted to 8.2 using 0.2 M NaOH. To 50 μL of oxidant solution, 10 μL of sample was added. The mixture was held at room temperature for 1 min before 2 μL of acetic acid was added to quench the reaction. Finally, the sample was transferred to a microvial for HPLC-MS analysis.

### 3.5. HPLC-HRMS Analysis

HPLC-HRMS analysis was performed on an UltiMate 3000 liquid chromatograph (Dionex, Sunnyvale, CA, USA) coupled to a Q Exactive Focus mass spectrometer (Thermo Fisher Scientific, San Jose, CA, USA). A reversed-phase Agilent Zorbax Eclipse Plus C18 column (2.1 × 150 mm, 1.8 μm particle size) was used for separation. All target compounds were chromatographed at 30 °C with a mobile phase consisting of formic acid in water (0.1% *v*/*v*, A) and formic acid in acetonitrile (0.1% *v*/*v*, B) at a flow rate of 0.3 mL/min. Gradient elution was applied: 2% B for 3 min, to 20% B in 2 min, to 50% B in 2 min, to 100% B in 0.1 min, hold for 2.9 min, to 2% B in 0.1 min, hold for 2.9 min.

All target compounds were simultaneously analyzed using the full-scan and PRM modes of HPLC−HRMS. Mass spectrometry conditions: positive and negative ion modes, scanning range of 50–750 *m*/*z*, resolution 35,000. The automatic gain control (AGC) was set to 5e^4^, and the maximum injection time (MIT) was set to automatic. The isolation window was set to 3.0 *m*/*z*. Mass tolerance was 5 ppm.

### 3.6. HPLC-MS/MS (MRM) Analysis

The HPLC–MS/MS (MRM) analysis was performed on an Agilent 1290 Infinity II HPLC system coupled to a triple quadrupole spectrometer (6495C, Agilent, Santa Clara, CA, USA). A reversed-phase Agilent Zorbax Eclipse Plus C18 column (2.1 × 150 mm, 1.8 μm particle size) was used for separation. All target compounds were chromatographed at 30 °C with a mobile phase consisting of formic acid in water (0.1% *v*/*v*, A) and formic acid in acetonitrile (0.1% *v*/*v*, B) at a flow rate of 0.3 mL/min. Gradient elution was applied: 2% B for 3 min, to 20% B in 2 min, to 25% B in 2 min, to 100% B in 0.1 min, hold for 2.9 min, to 2% B in 0.1 min, hold for 2.9 min.

All target compounds were quantified in multiple reaction monitoring (MRM) mode using a jet stream electrospray ionization source in positive ion mode and negative mode. Parameters are listed in Table S1. Ionization source parameters were as follows: gas temperature, 250 °C; gas flow rate, 11 L/min; nebulizer gas pressure, 35 psi; sheath gas temperature, 300 °C; sheath gas flow rate, 12 L/min; capillary voltage, 3500 V; nozzle voltage, 0 V; fragmentation voltage, 166 V.

### 3.7. Method Validation

Specificity was evaluated by comparing multiple reaction monitoring (MRM) chromatograms of blank plasma with the corresponding spiked plasma samples.

Linearity was assessed by reference solutions ranging from LOQs to 50 ng/mL or 75 ng/mL. Calibration curves are in the form of y = Ax + B, where y represents the peak area of the main oxidation product and x represents the concentration of spiked PSTs. A linear least-squares regression analysis was conducted to determine the slope, intercept, and correlation coefficient (r^2^) to demonstrate the linearity of this method. LODs and LOQs were determined to be ~3 times and 10 times the signal-to-noise ratios (S/N) of the target peak area response in the blank plasma.

Accuracy was calculated in terms of recoveries (R%). The intra-day and inter-day accuracy and precision were evaluated by spiking the human plasma with two levels (LOQ and 50 ng/mL) of PSTs reference, performing detection for four consecutive days and six replicates per day, respectively. The stability of the major oxidation product was assessed by storing the reference solution (LOQs and 50 ng/mL) after vanadium pentoxide oxidation at 15 °C for 48 h, and comparing the response intensities before and after incubation.

### 3.8. Analysis of Samples from the First Trial OPCW Biotoxin Proficiency Test

The aim of the First Trial OPCW Biotoxins Proficiency Test (ToxPT) is to establish and maintain a recognized and transparent methodology for the continued assessment of the technical competence of the participating laboratories that are seeking future designation for biotoxin analysis or retention of future designation status. The ToxPT is conducted once per calendar year, and the laboratories must demonstrate that they have maintained their capability by successfully participating in every relevant ToxPT organized by the OPCW Secretariat. The established method was applied to the analysis of six samples (TBT011-TBT016), comprising three liquid and three aqueous samples, obtained from the First Trial OPCW Biotoxin Proficiency Test held in 2024.

## 4. Conclusions

In this study, we present a robust HPLC-MS/MS (MRM) method for PSTs screening based on vanadium pentoxide-mediated oxidation. This oxidant efficiently converts a broad spectrum of fourteen kinds of PSTs into characteristic major products (P1, P2, P4, P5, P6, P7) via cleavage of the C4-C12 bond, accompanied by loss of four hydrogen atoms and, where relevant, elimination of N-1-hydroxy or C-11 sulfonate groups. These unique markers enable unambiguous differentiation of PST subgroups (STX/neoSTX, GTX1/2/3/4, GTX5/6, and C1/2), while product distribution patterns further distinguish dcSTX/dcneoSTX from GTX5/6 and dcGTX2/3 from C1/2. Compared with periodate, vanadium pentoxide offers superior product selectivity for ten toxins among the fourteen toxins. This method enables qualitative analysis of PSTs in plasma samples. Moreover, the successful detection of STX and/or neoSTX in samples from the First Trial of the OPCW Biotoxin Proficiency Test confirmed its practical applicability.

## Figures and Tables

**Figure 1:**
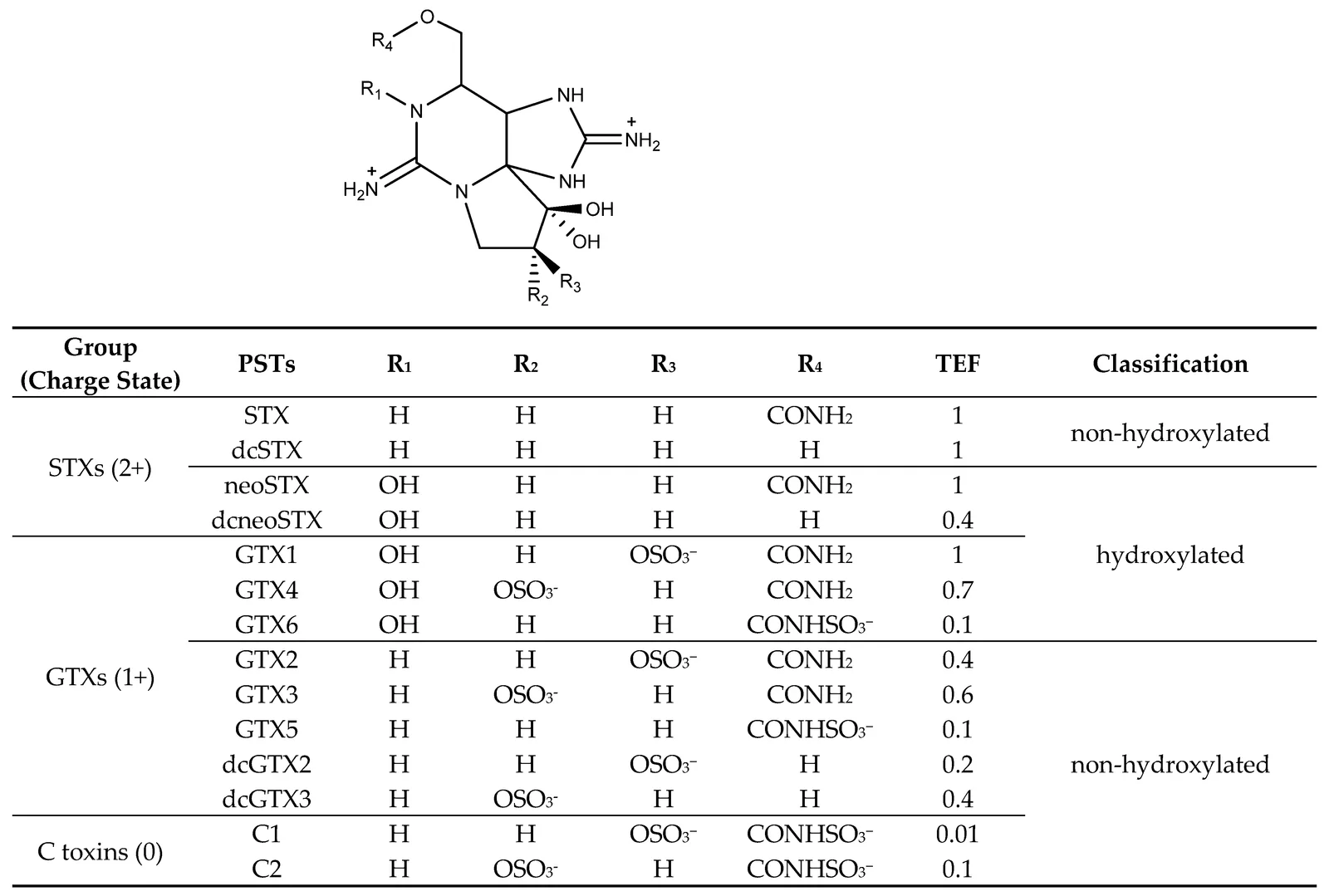
Chemical structures of fourteen paralytic shellfish toxins (PSTs), grouped by classification and charge state. TEF: Toxicity Equivalence Factor [5].

**Figure 2:**
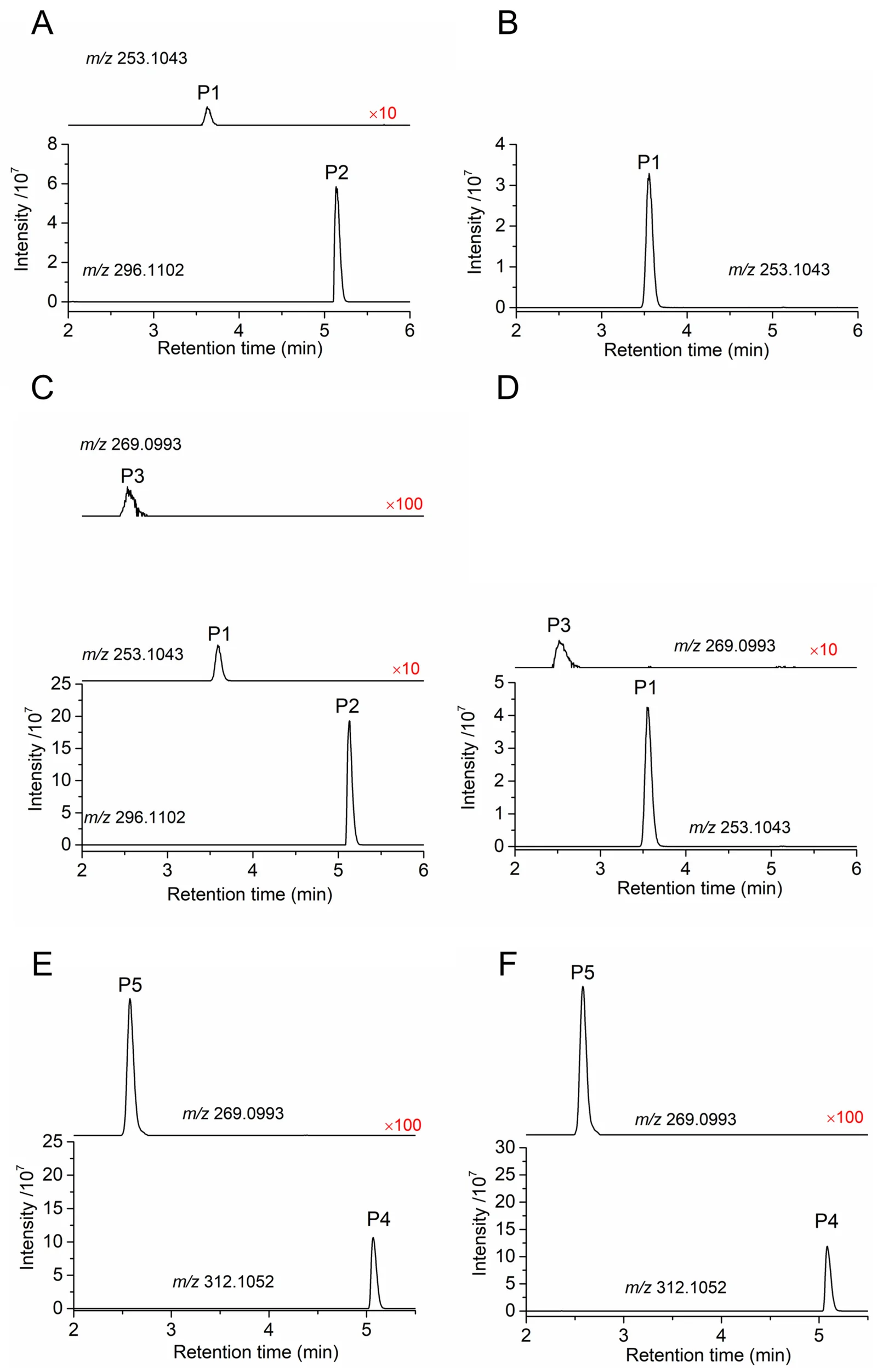

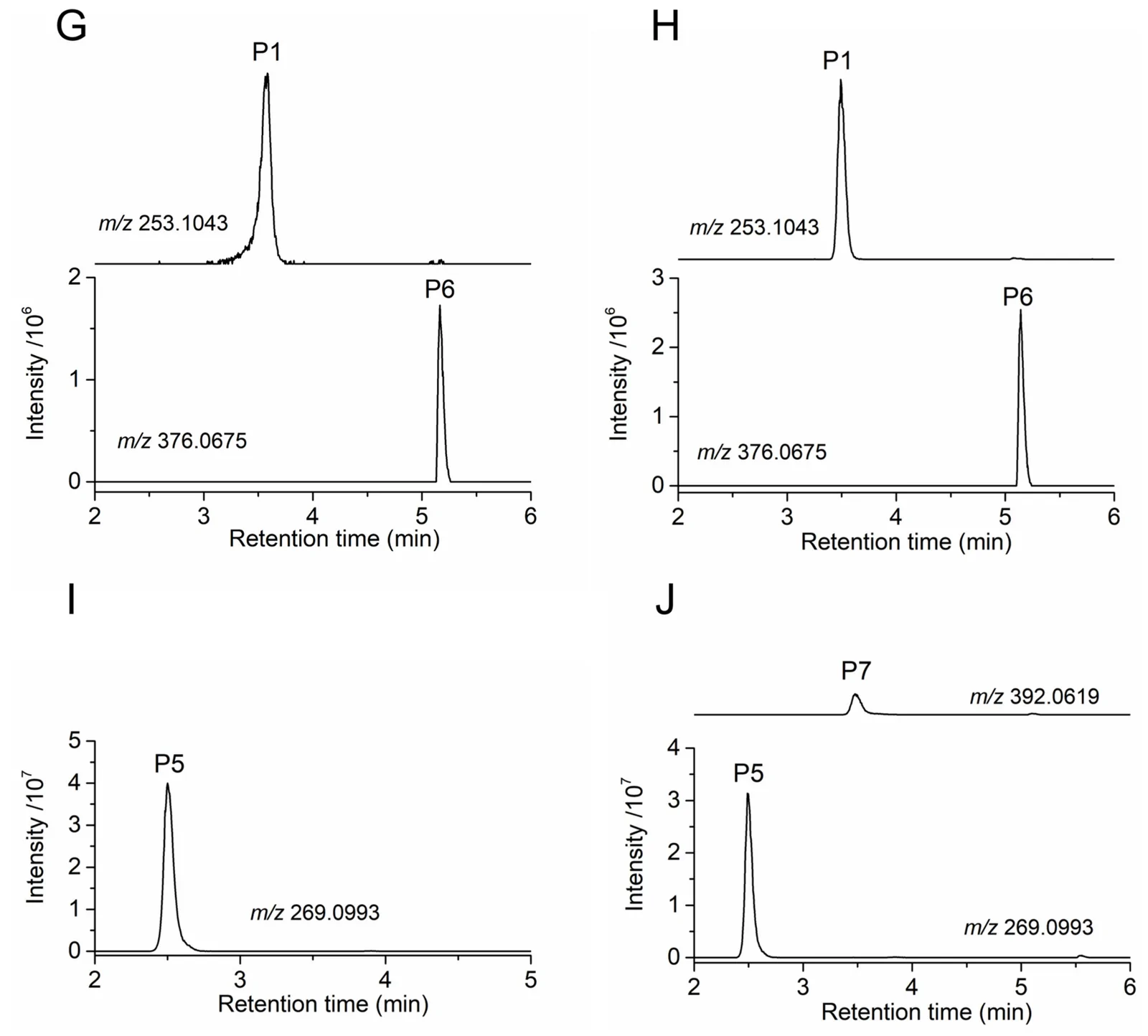
Total ion chromatogram from HPLC−HRMS analysis of STX (**A**), dcSTX (**B**), neoSTX (**C**), dcneoSTX (**D**), GTX1/4 (**E**), GTX2/3 (**F**), GTX5 (**G**), GTX6 (**H**), dcGTX2/3 (**I**), and C1/2 (**J**) reference solution after vanadium pentoxide oxidation.

**Figure 3:**
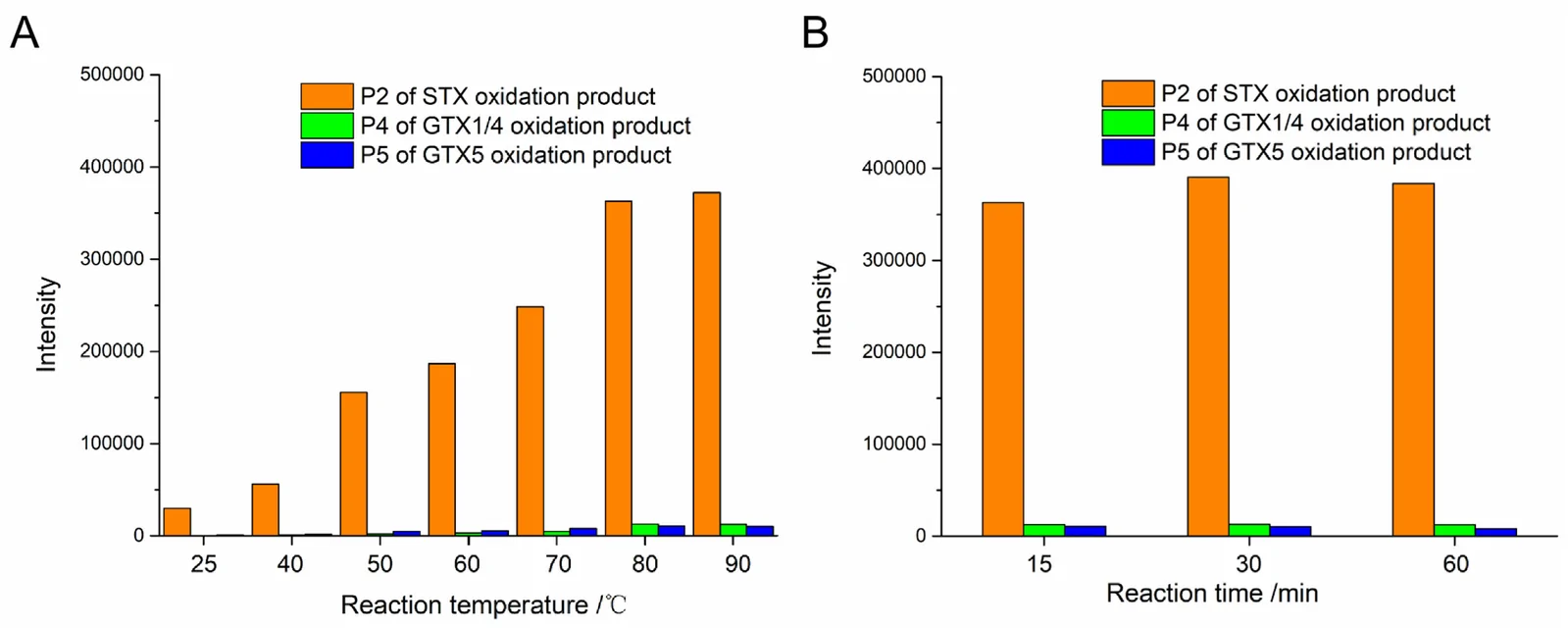
Optimization of reaction temperature (**A**) and reaction time (**B**) for STX, GTX1/4, and GTX5 in vanadium pentoxide oxidation reaction.

**Figure 4:**
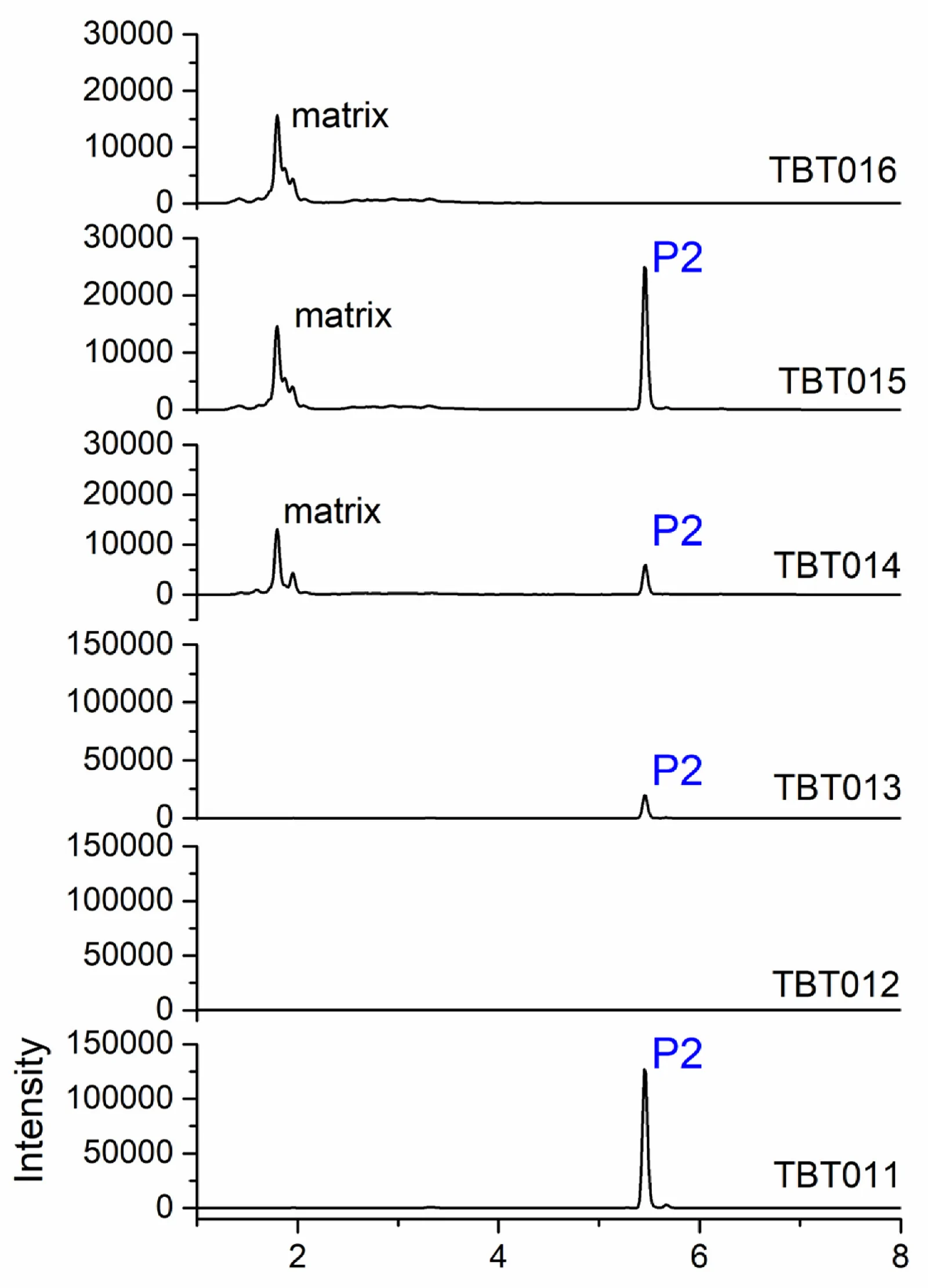
The HPLC-MS/MS (MRM) analysis of six samples from the First Trial OPCW Biotoxin PT after oxidation by vanadium pentoxide.

**Table 1:** Linear dynamic ranges, calibration curves, LODs and accuracy (R%), and precision (intra- and inter-day RSD) of PSTs based on vanadium pentoxide oxidation and HPLC-MS/MS analysis.

Analytes	Regression Equation	Correlation Coefficient (r^2^)	Linear Ranges ng/mL	LOD (ng/mL)	LOQ (ng/mL)	(R%)/RSD Intra-Day (*n* = 6)		(R%)/RSD Inter-Day (*n* = 4)	
						LOQ	50 ng/mL	LOQ	50 ng/mL
STX	y = 6849x + 312	0.9986	0.1~50	0.3	1.0	96.6/0.4	100.3/5.4	99.8/4.5	96.5/0.3
dcSTX	y =6377x + 254	0.9958	0.2~50	0.3	1.0	96.6/0.9	94.5/9.8	99.4/4.0	101.4/8.7
neoSTX	y = 7496x + 341	0.9996	0.1~50	0.3	1.0	94.7/5.6	96.2/3.6	96.5/2.6	93.6/6.2
dcneoSTX	y = 3370x + 159	0.9999	0.2~50	0.3	1.0	93.5/4.7	94.2/0.6	97.2/5.2	93.8/9.3
GTX1/4	y = 528x + 107	0.9999	0.5~75	1.0	3.0	91/3.7	95.6/0.9	98.1/10	95/6.9
GTX2/3	y = 510x + 12	0.9995	0.5~75	1.0	3.0	92.6/0.6	102.2/8.1	100/10.4	91.9/2.4
GTX5	y = 573x + 16	0.9989	0.5~50	0.5	1.5	96/3.3	97.7/2.2	97.9/2.8	96.1/3.6
GTX6	y = 391x + 241	0.9984	0.5~75	0.5	1.5	93.2/3.2	101.9/7.7	102.6/9.1	96.4/2.6
dcGTX2/3	y = 156x + 30	0.9998	0.5~75	1.0	3.0	96.3/7.9	90.4/1.6	94.9/2.0	89.3/5.1
C1/2	y = 123x + 143	0.9967	0.5~75	1.5	5.0	100.6/4.9	98.2/7.5	103.4/3.9	92.9/8.6

## Data Availability

The original data presented in the study are included in the article/Supplementary Material; further inquiries can be directed to the corresponding author.

## Supplementary Materials

The following supporting information can be downloaded at: https://www.mdpi.com/article/10.3390/md24100343/s1, Figure S1: Reaction scheme for vanadium pentoxide oxidize fourteen PSTs; Figure S2: MS/HRMS spectrum of the products derived from fourteen PSTs reference after vanadium pentoxide oxidation; Figure S3: Influence of pH of the reaction buffer in vanadium pentoxide oxidation on the yield of main products of fourteen PSTs; Figure S4: Total ion chromatogram from HPLC−MS/MS analysis of fourteen PSTs reference after peridoate oxidation and vanadium pentoxide oxidation; Figure S5: Calibration plots of fourteen PSTs concentration against mass spectrum intensity for their main oxidation products after vanadium pentoxide oxidation. Table S1: Optimized ion transition parameters for PSTs oxidation products; Table S2: Reaction products after oxidation with vanadium pentoxide; Table S3: Stability of main PSTs oxidation products after vanadium pentoxide oxidation; Table S4: Comparison of limits of detection for PSTs in plasma between the present method and HILIC-MS/MS approach.

## Abbreviations

The following abbreviations are used in this manuscript:

HILIC–MS/MS	Hydrophilic interaction liquid chromatography–tandem mass spectrometry
HPLC-HRMS	High-performance liquid chromatography–tandem high-resolution mass spectrometry
LOD	Limits of detection
LOQ	Limits of quantitation
PSTs	The paralytic shellfish toxins
STX	Saxitoxin
