# Supplementary material for: Vanadium Pentoxide–Mediated Oxidation Coupled with HPLC-MS/MS for Broad-Spectrum Screening of Paralytic Shellfish Toxins in Plasma
Source: Mar Drugs. 2026 Sep 29;24(10):343. doi: 10.3390/md24100343 (PMC13706285; doi:10.3390/md24100343)
Supplement: Supplementary file 1 [file marinedrugs-24-00343-s001.zip › marinedrugs-4545404-supplementary.pdf]

Contents

Figure S1 4

Figure S2 13

Figure S3 14

Figure S4 16

Figure S5 17

Table S1 18

Table S2 19

Table S3 20

Table S4 21

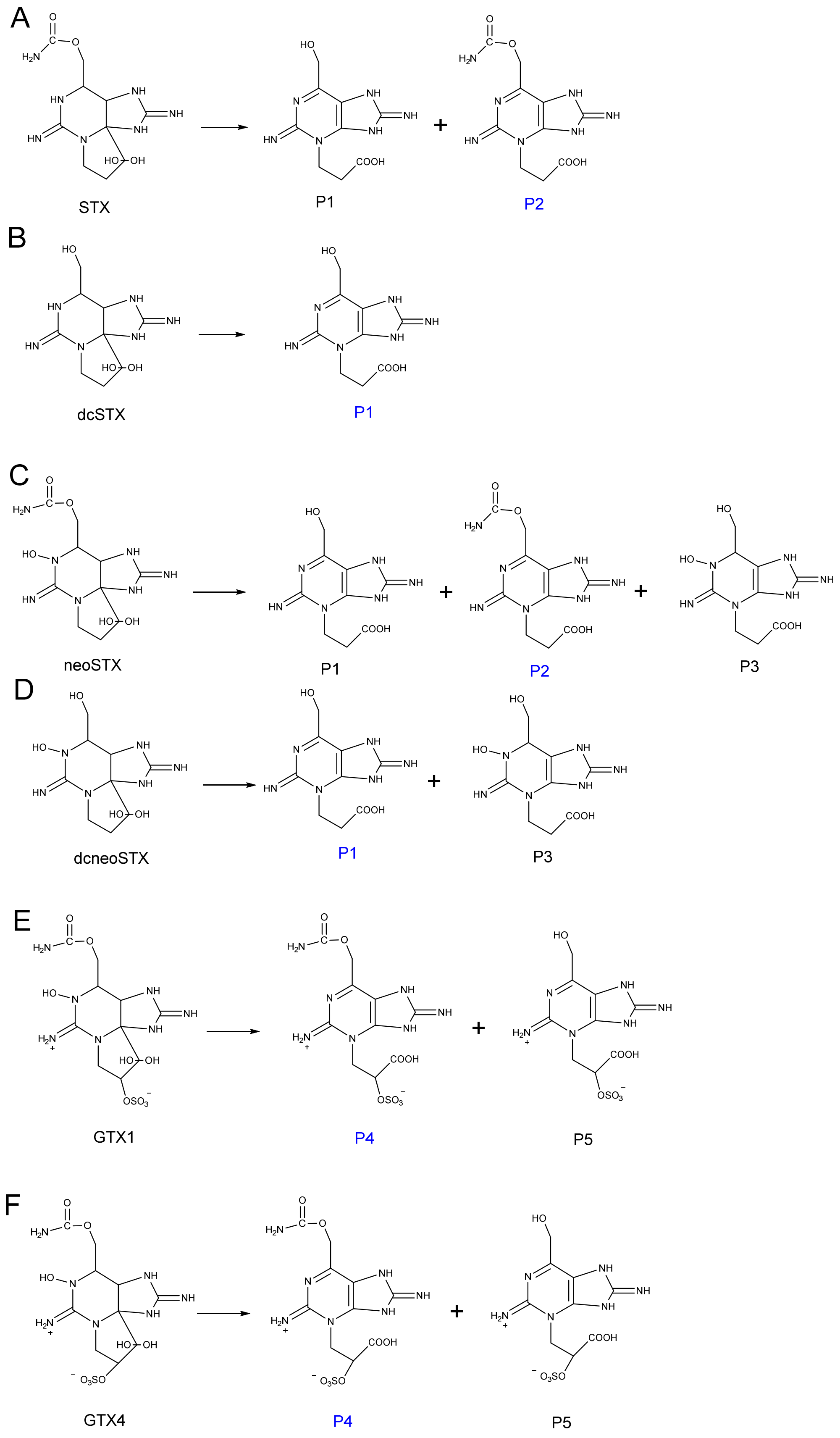
A
STX
P1
P2
B
dcSTX
P1
C
neoSTX
P1
P2
P3
D
dcneoSTX
P1
P3
E
GTX1
P4
P5
F
GTX4
P4
P5

G

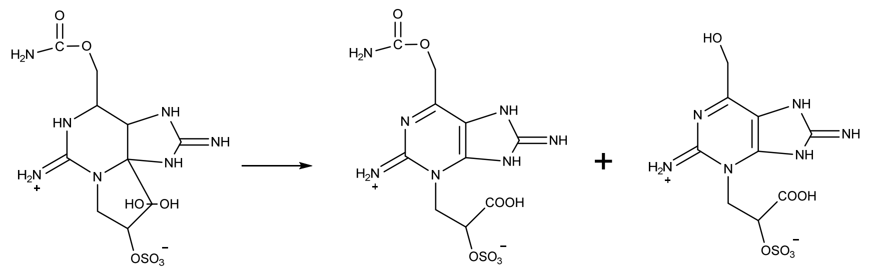

GTX2 P4 P5

H

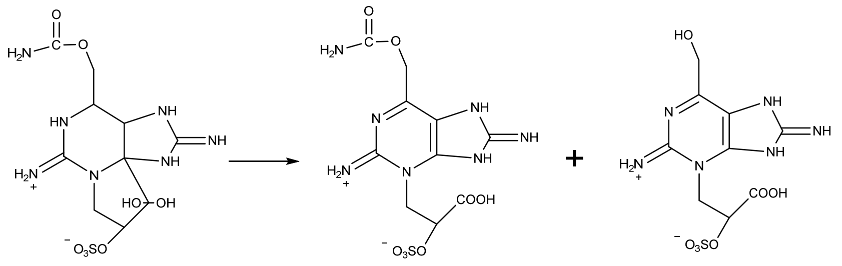

GTX3 P4 P5

I

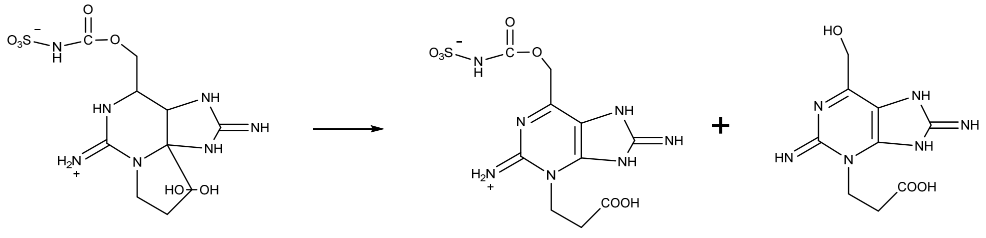

GTX5 P6 P1

J

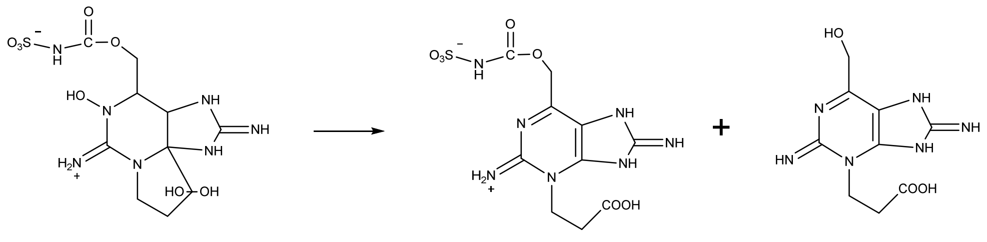

GTX6 P6 P1

K

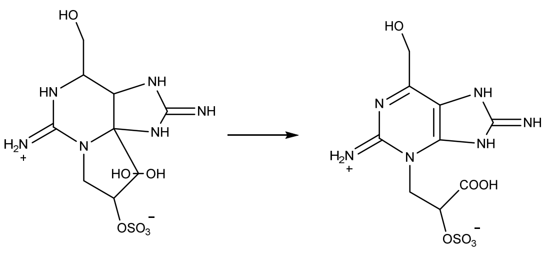

dcGTX2 P5

L

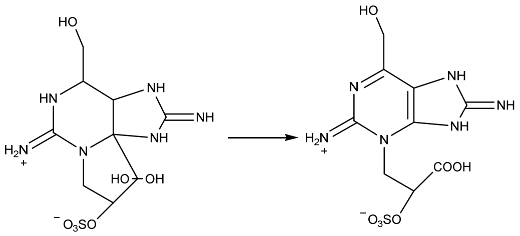

dcGTX3 P5

M

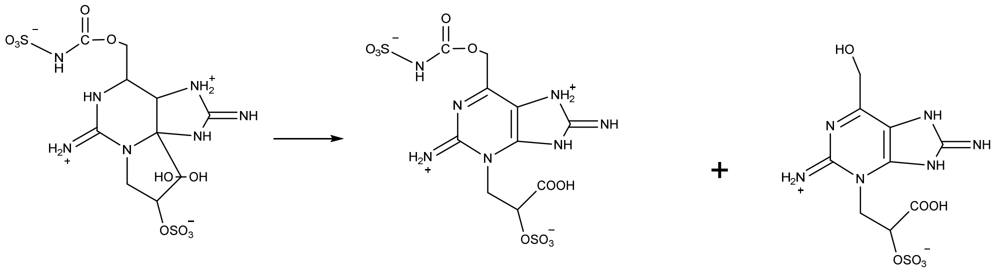

C1 P7 P5

N

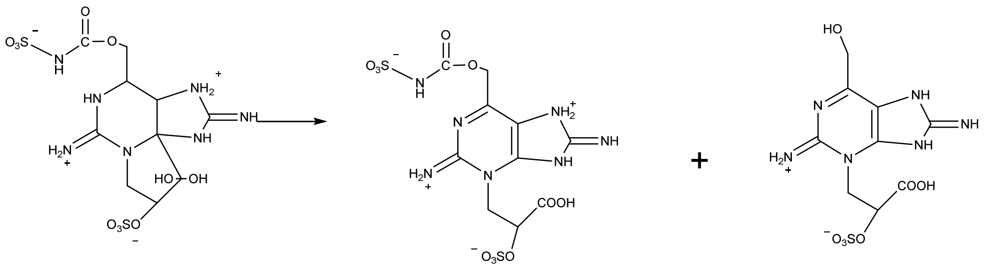

C2 P7 P5

Figure S1. Reaction scheme for the oxidation of STX (A), dcSTX (B), neoSTX (C), dcneoSTX (D), GTX1 (E), GTX4 (F), GTX2 (G), GTX3 (H), GTX5(I), GTX6 (J), dcGTX2 (K), dcGTX3 (L), C1 (M) and C2 (N) by vanadium pentoxide. The blue products represent main products.[32-37]

A STX oxidation

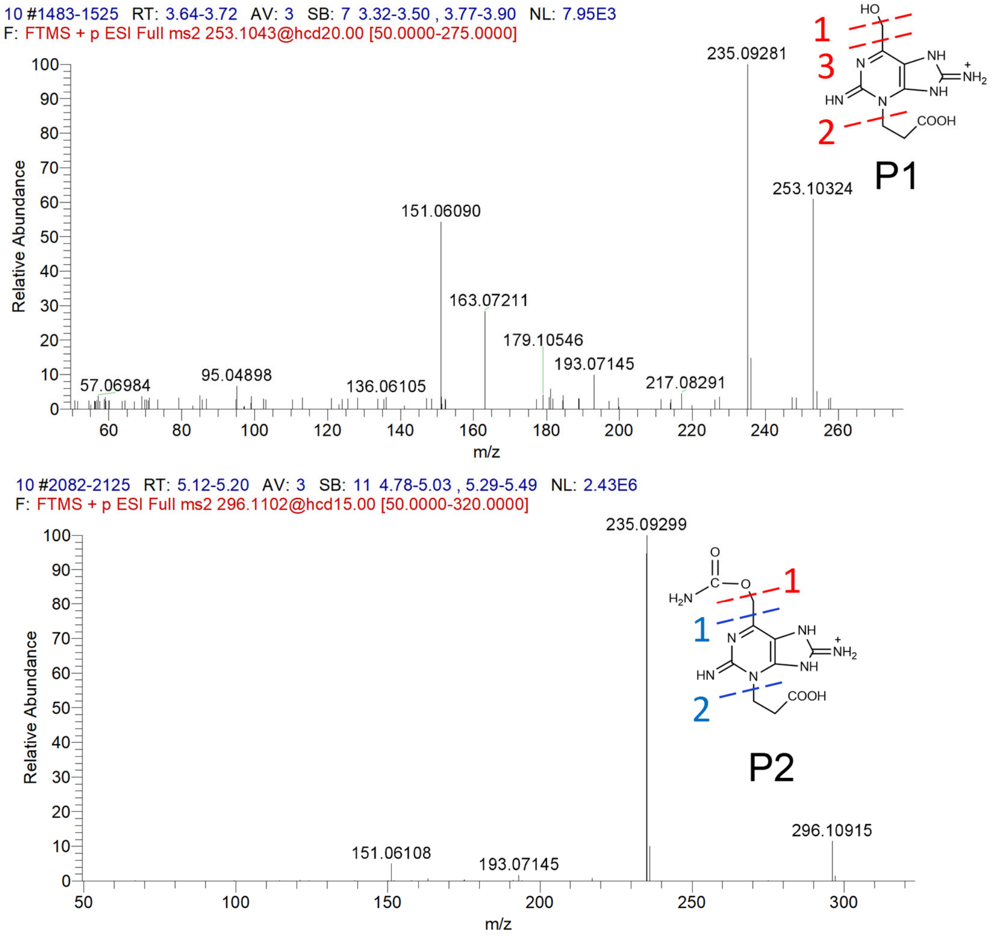

B dcSTX oxidation

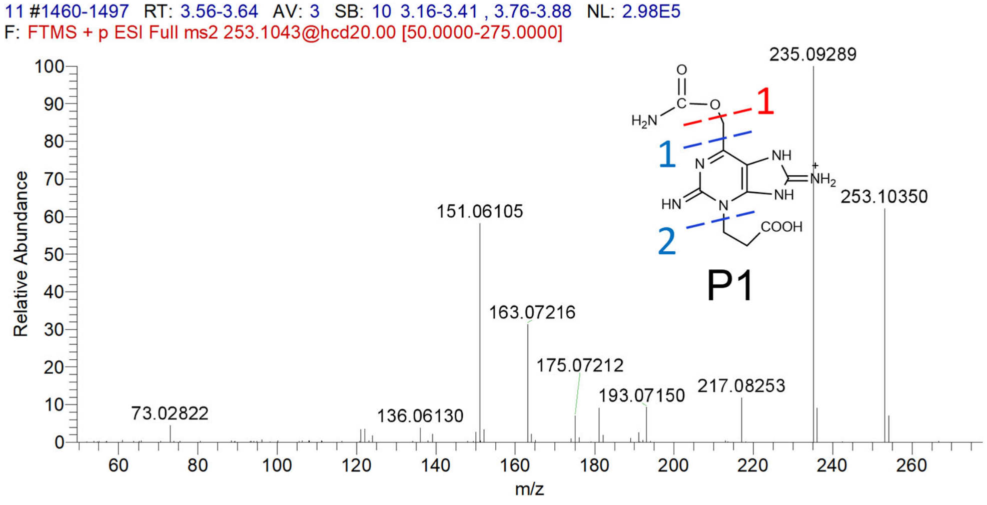

C neoSTX oxidation

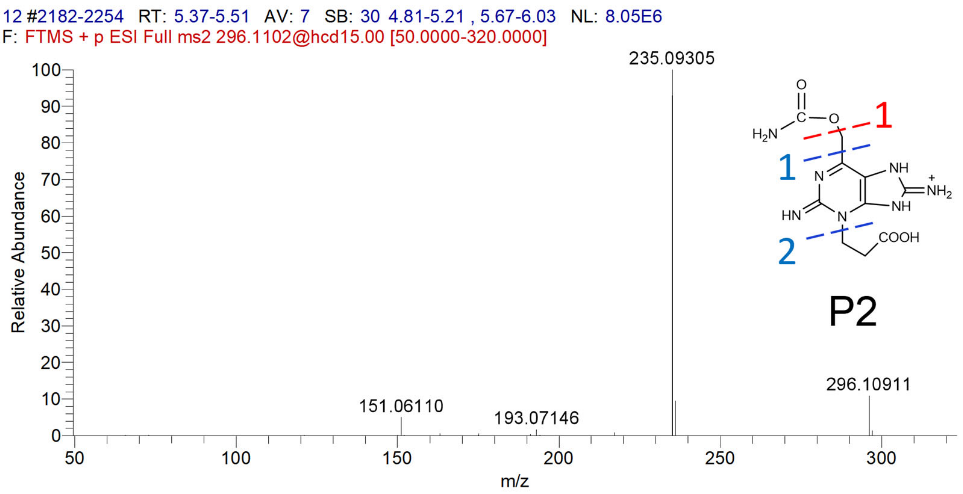

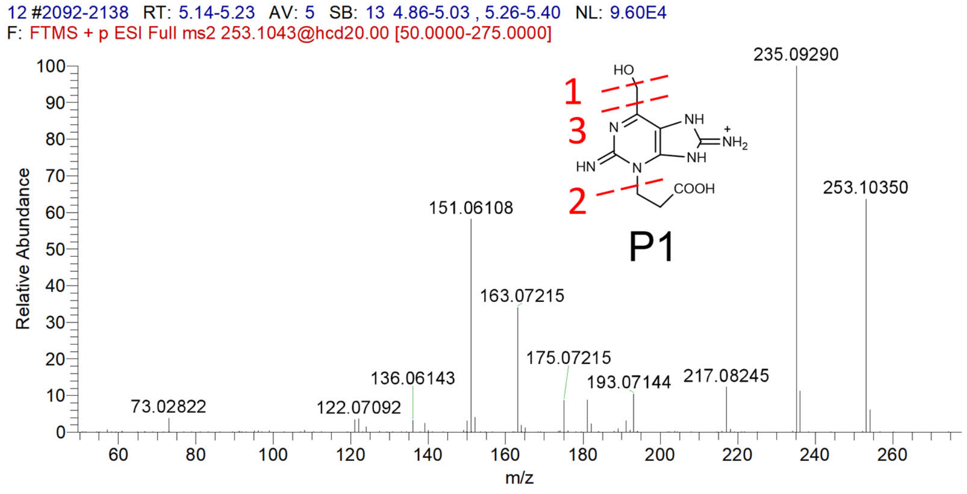

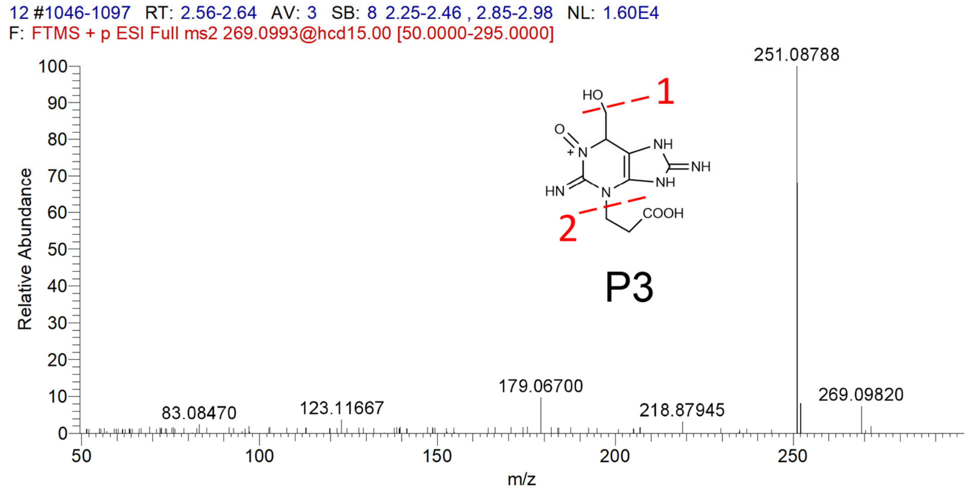

D dcneoSTX oxidation

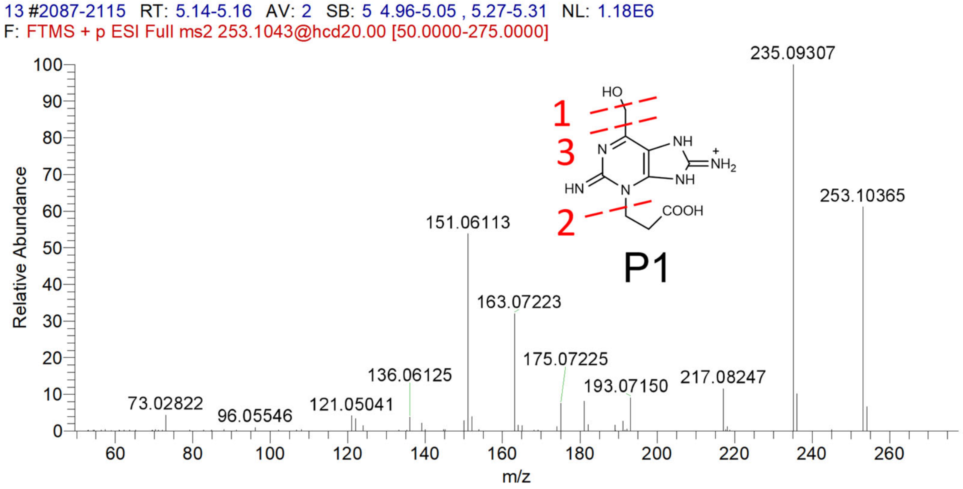

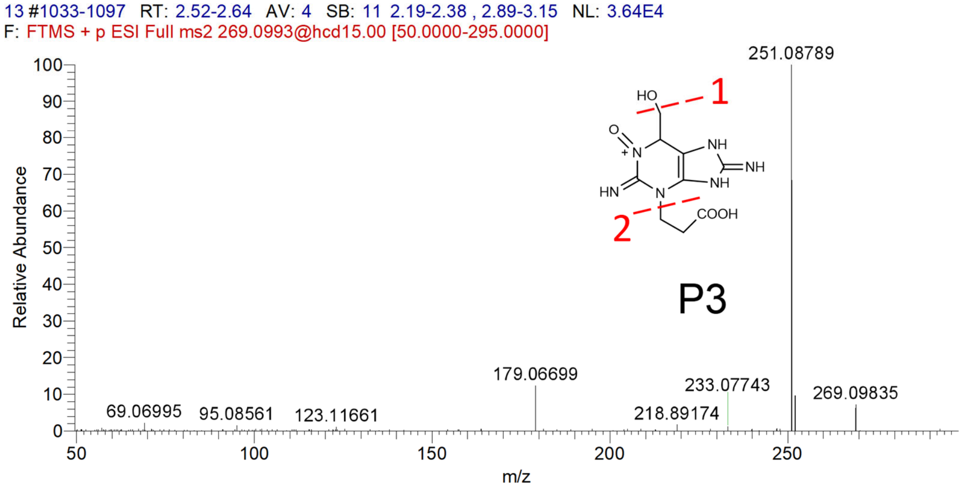

E GTX14 oxidation

positive ion mode

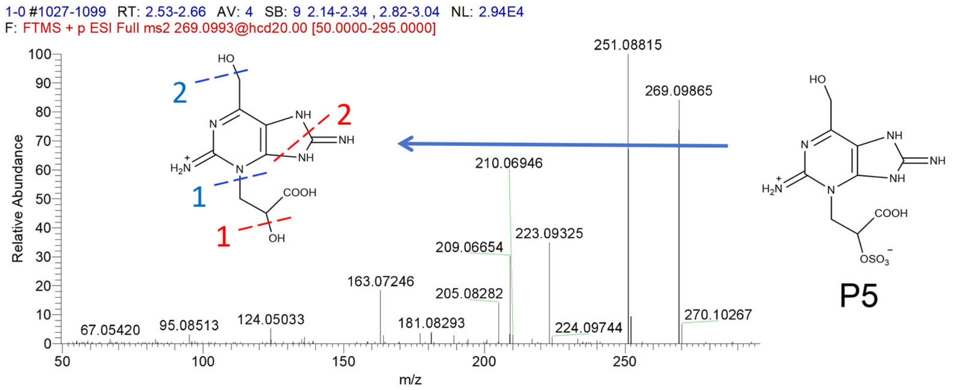

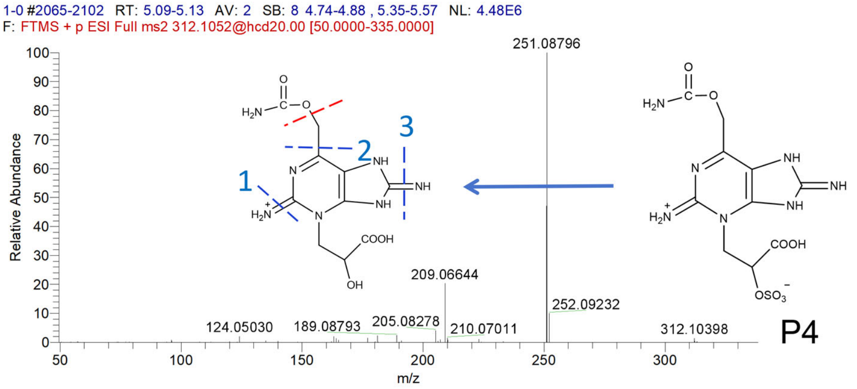

negative ion mode

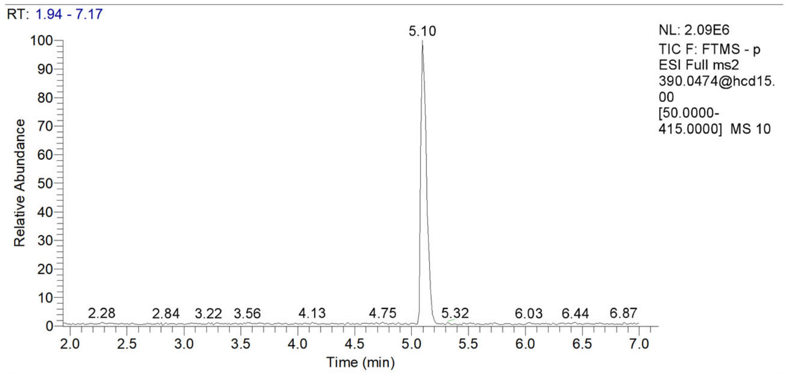

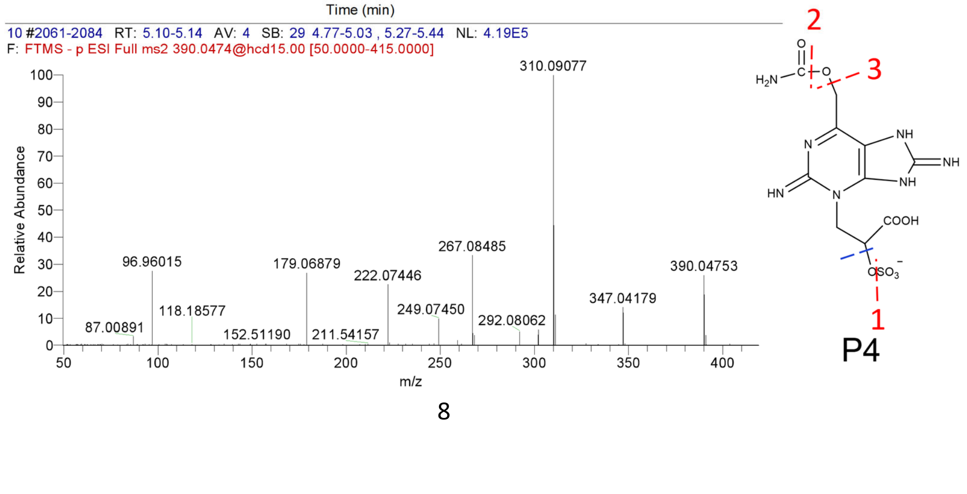

F GTX2/3 oxidation

positive ion mode

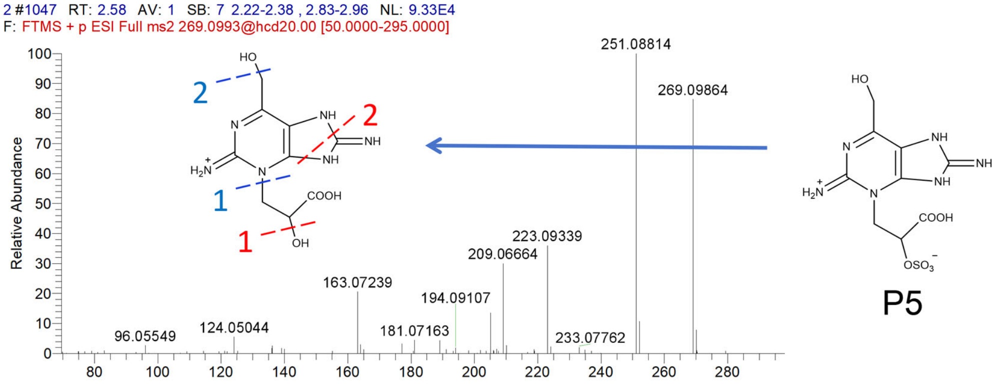

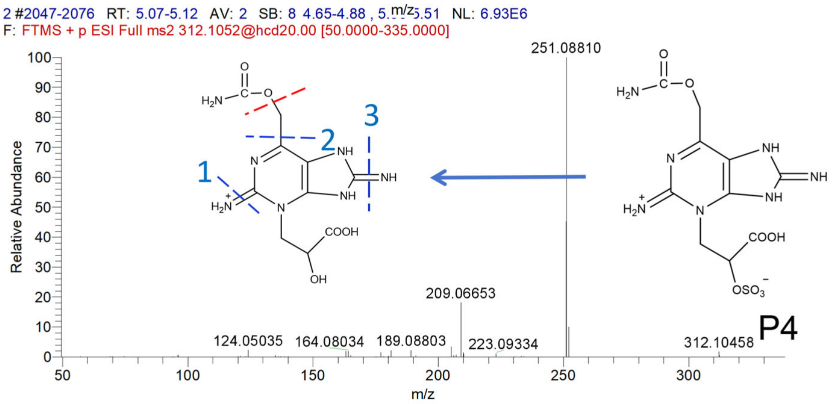

negative ion mode

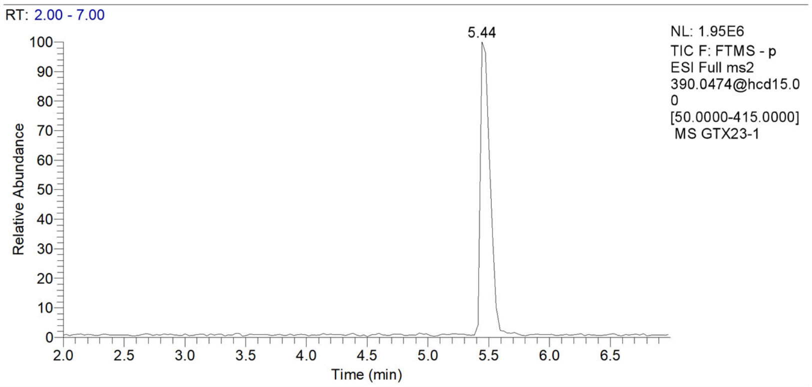

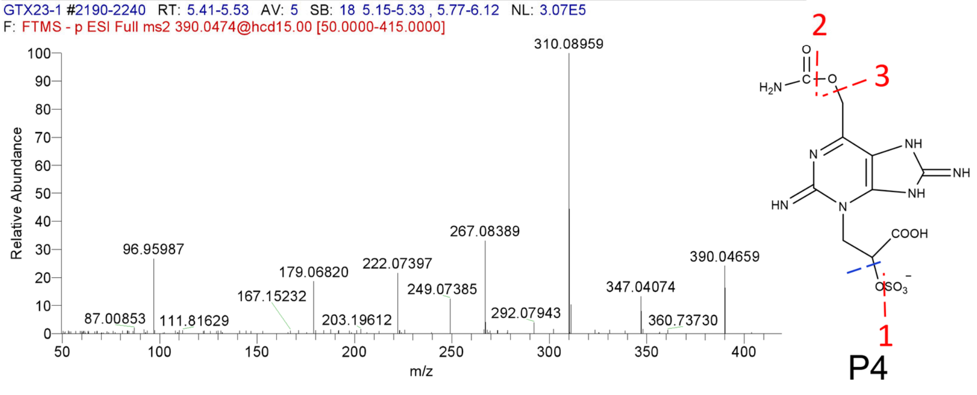

G

GTX5 oxidation

positive ion mode

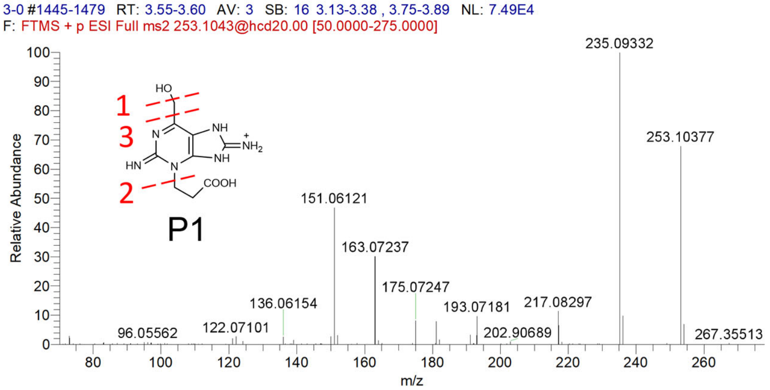

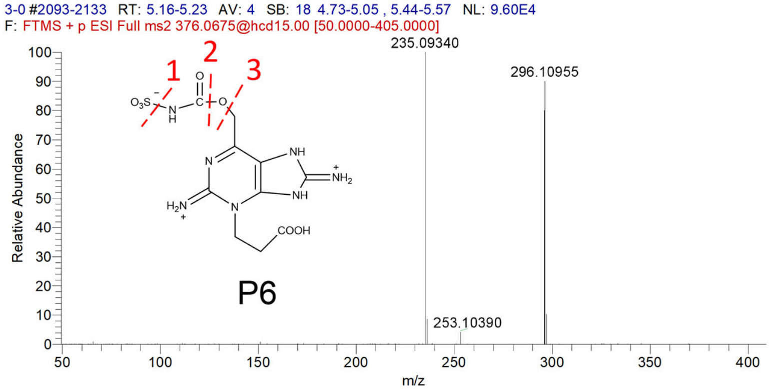

negative ion mode

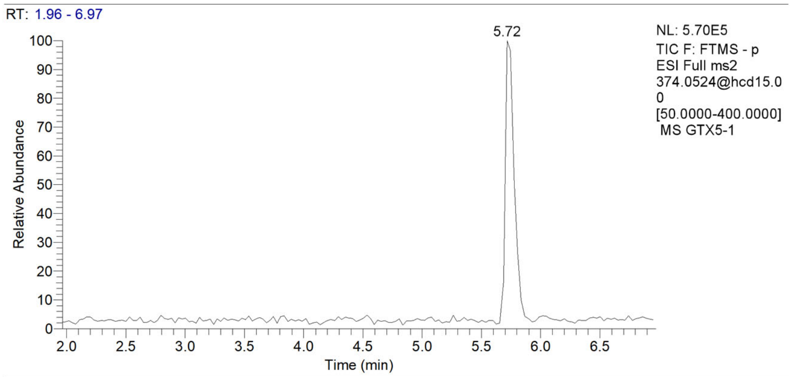

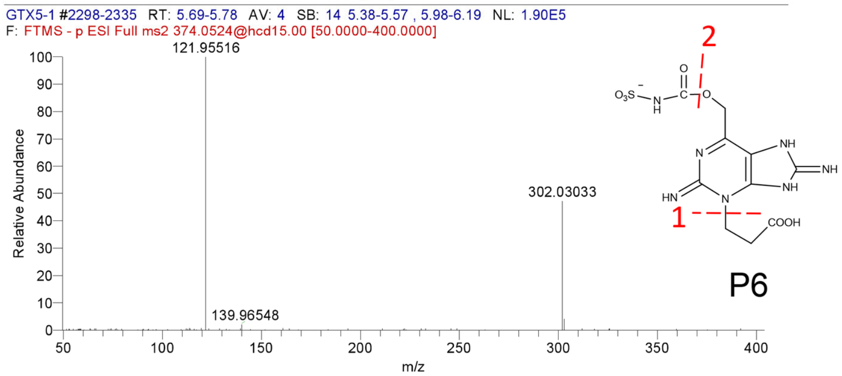

H GTX6 oxidation

positive ion mode

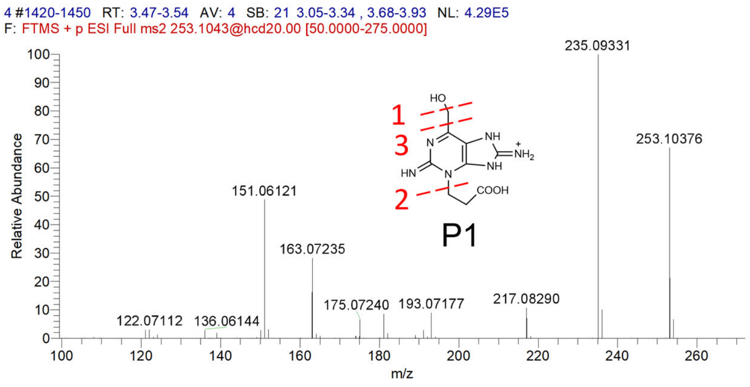

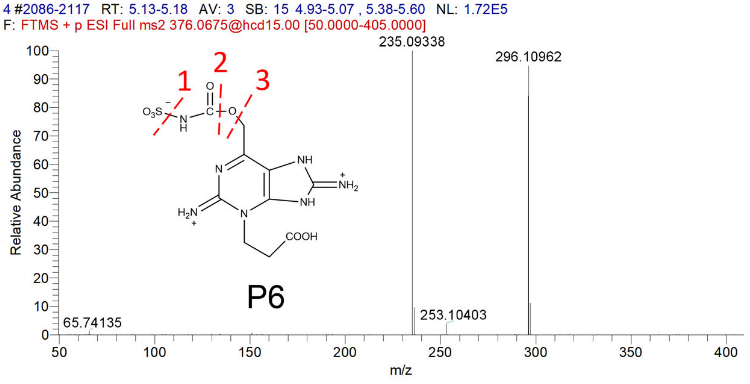

negative ion mode

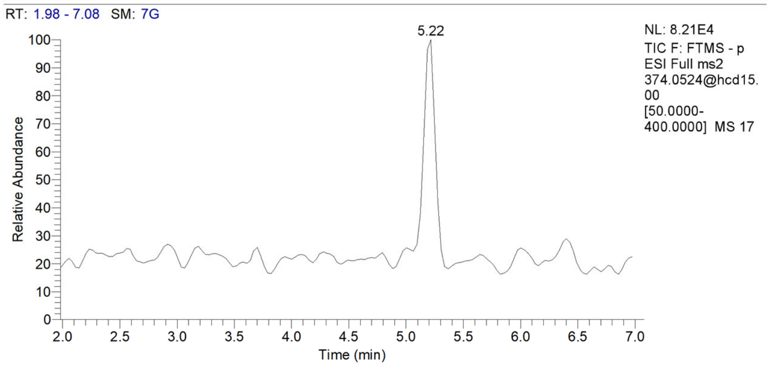

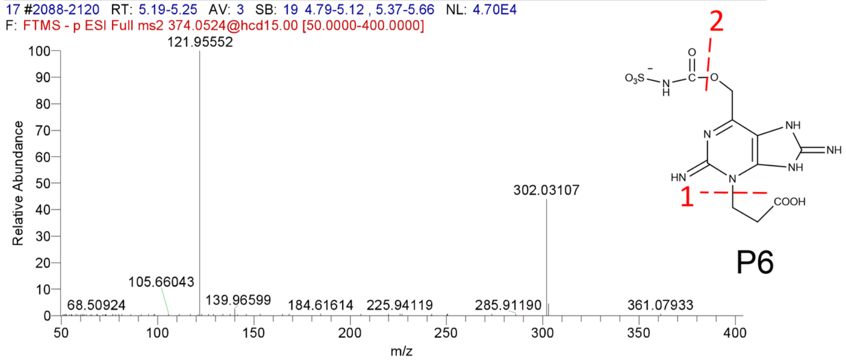

I dcGTX2/3 oxidation

positive ion mode

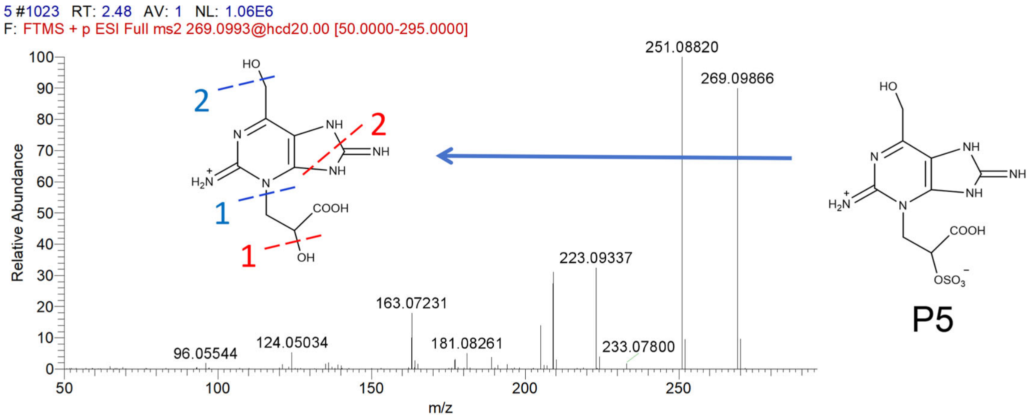

negative ion mode

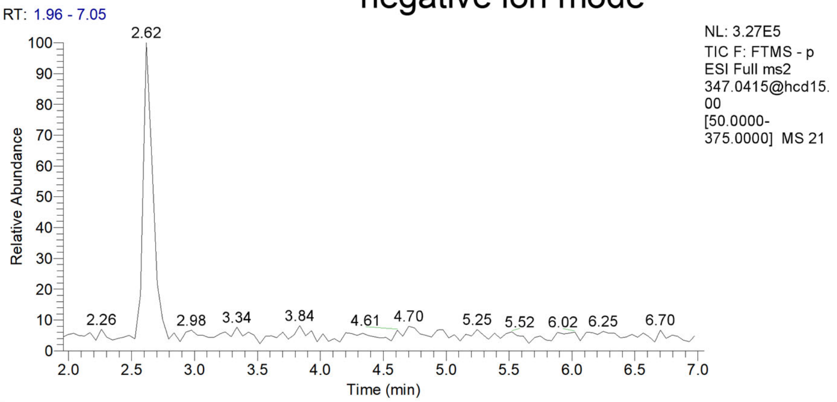

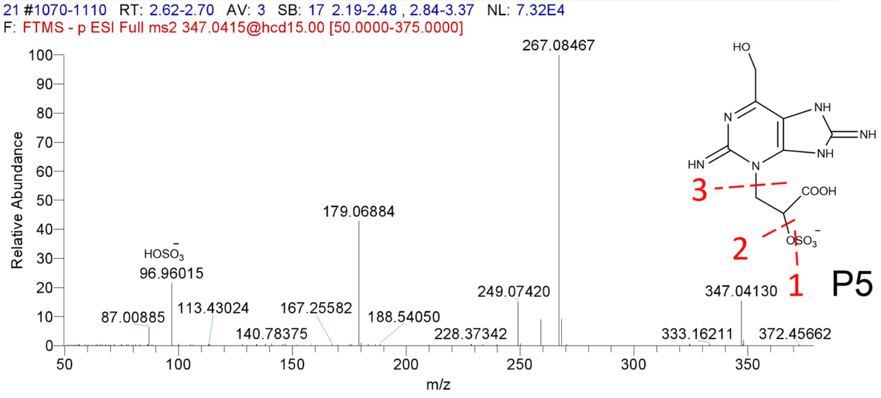

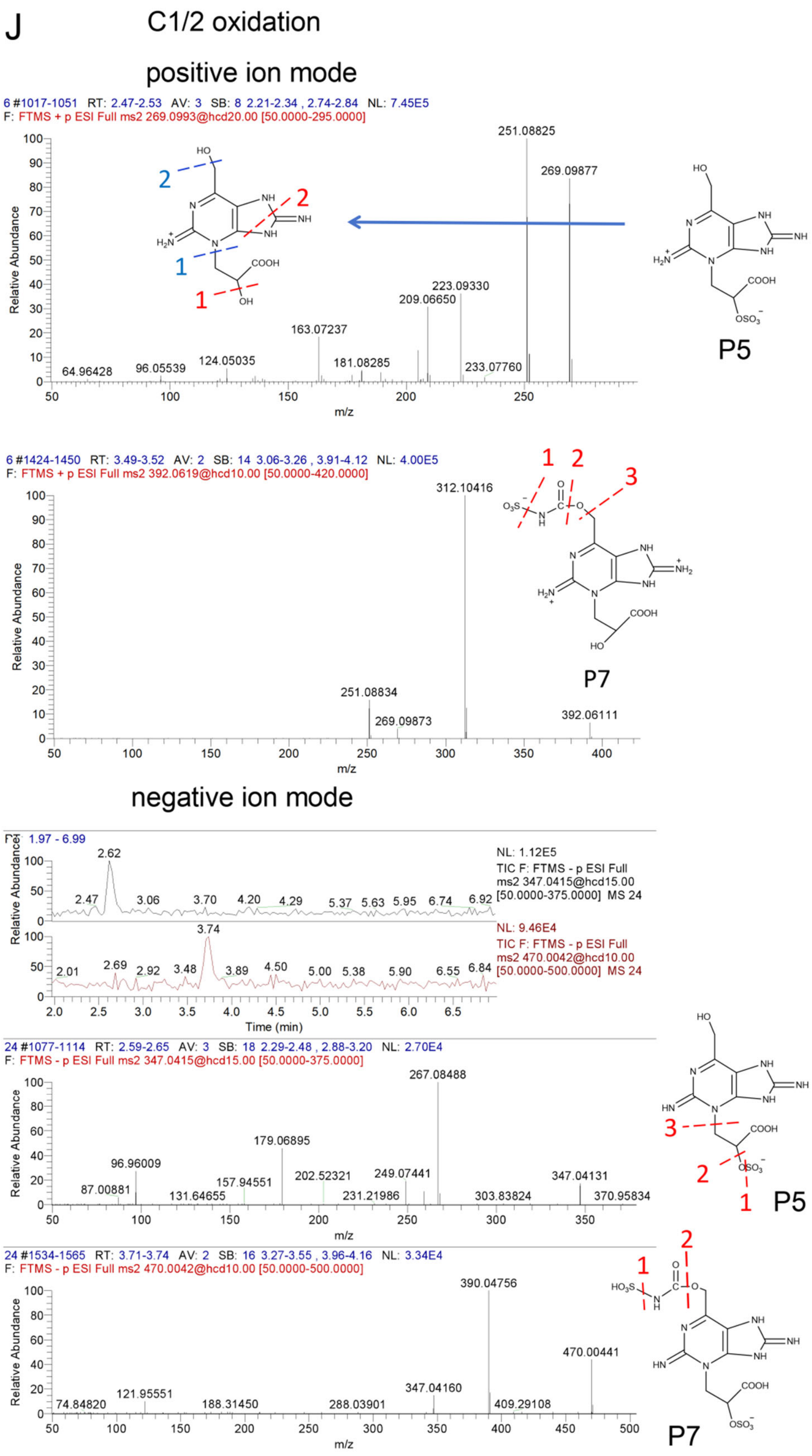

Figure S2. MS/HRMS spectrum of the products derived from STX (A), dcSTX (B), neoSTX (C), dcneoSTX (D), GTX1/4 (E), GTX2/3 (F), GTX5(G), GTX6 (H), dcGTX2/3 (I), and C1/2 (J) reference after vanadium pentoxide oxidation.

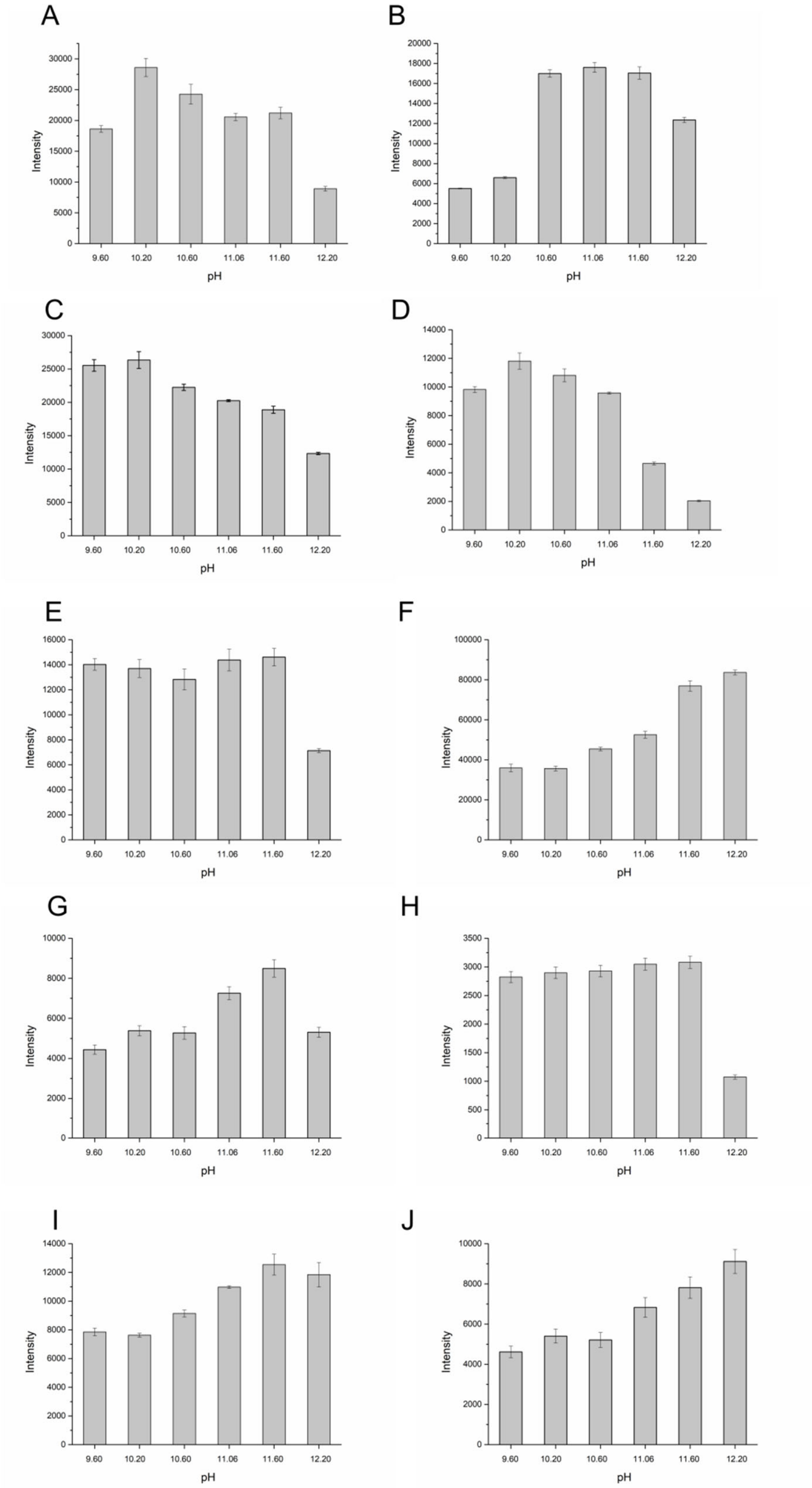

Figure S3. Influence of pH of the reaction buffer in vanadium pentoxide oxidation on the yield of main products of STX (A), dcSTX (B), neoSTX (C), dcneoSTX (D), GTX1/4 (E), GTX2/3 (F), GTX5(G), GTX6 (H), dcGTX2/3 (I), and C1/2 (J).

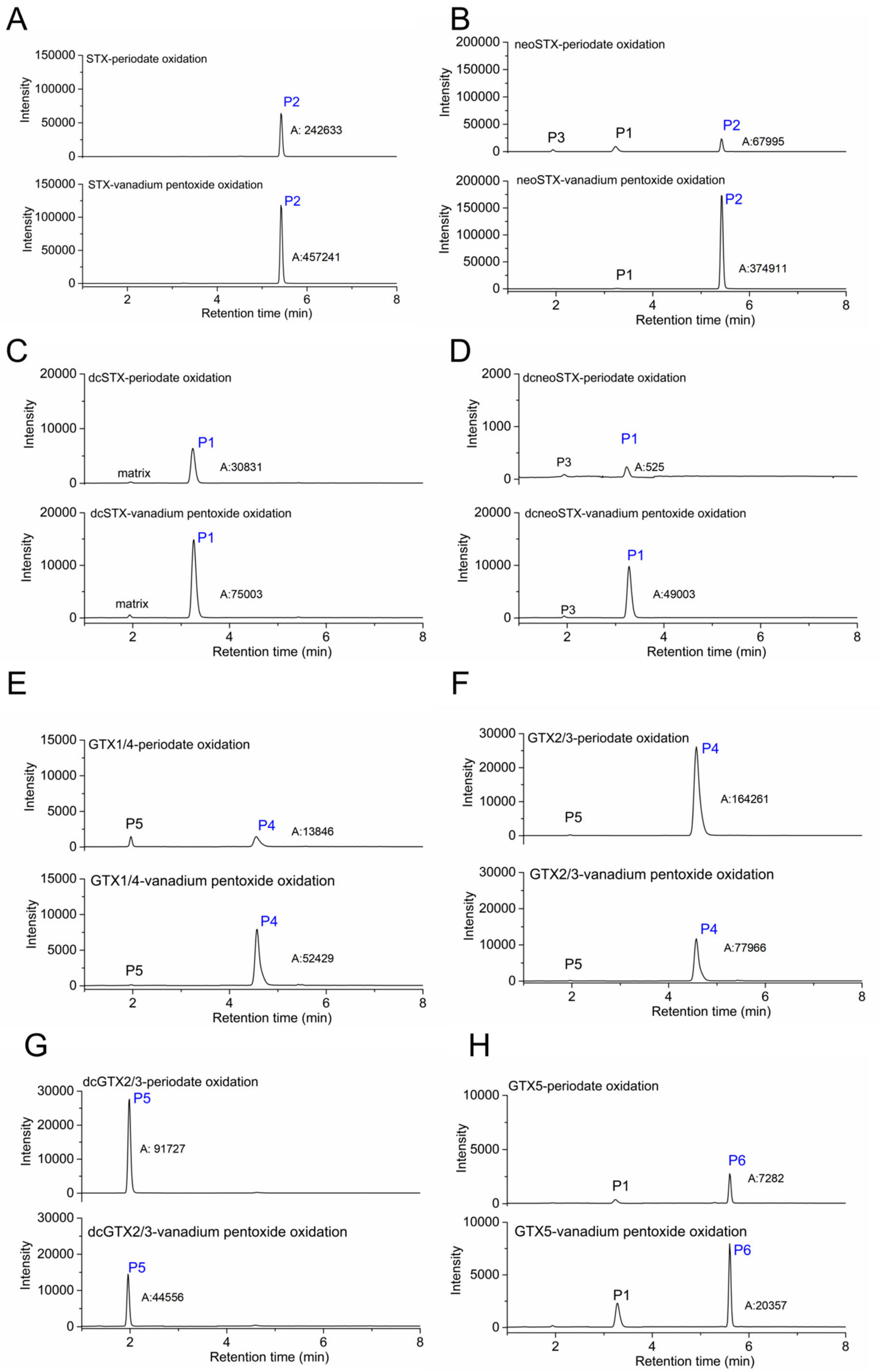
A
STX-periodate oxidation
P2
A: 242633
STX-vanadium pentoxide oxidation
P2
A:457241
B
neoSTX-periodate oxidation
P3
P1
P2
A:67995
neoSTX-vanadium pentoxide oxidation
P2
P1
A:374911
C
dcSTX-periodate oxidation
P1
matrix
A:30831
dcSTX-vanadium pentoxide oxidation
P1
matrix
A:75003
D
dcneoSTX-periodate oxidation
P1
P3
A:525
dcneoSTX-vanadium pentoxide oxidation
P1
P3
A:49003
E
GTX1/4-periodate oxidation
P5
P4
A:13846
GTX1/4-vanadium pentoxide oxidation
P4
P5
A:52429
F
GTX2/3-periodate oxidation
P4
P5
A:164261
GTX2/3-vanadium pentoxide oxidation
P4
P5
A:77966
G
dcGTX2/3-periodate oxidation
P5
A: 91727
dcGTX2/3-vanadium pentoxide oxidation
P5
A:44556
H
GTX5-periodate oxidation
P6
P1
A:7282
GTX5-vanadium pentoxide oxidation
P6
P1
A:20357
Intensity
Retention time (min)

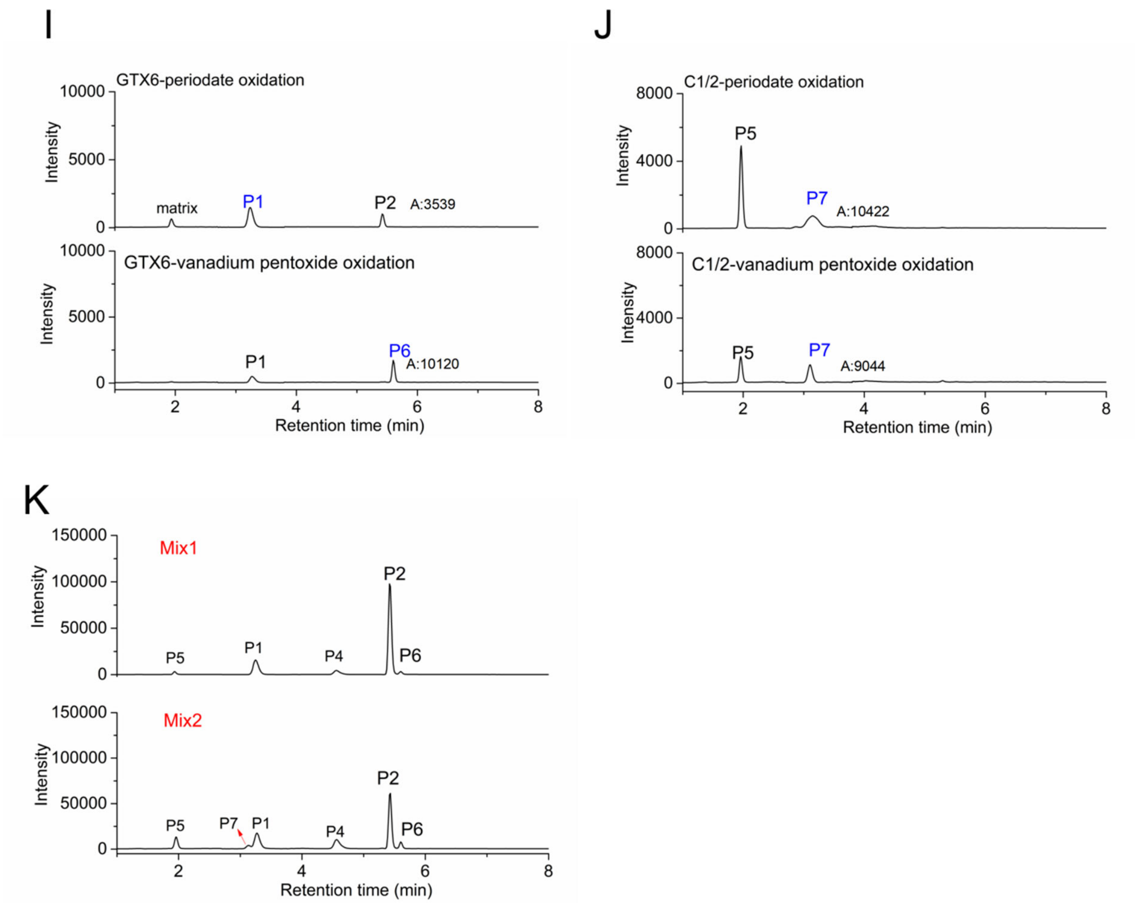

Figure S4. Total ion chromatogram from HPLC-MS/MS analysis of STX (A), neoSTX (B), dcSTX (C), dcneoSTX (D), GTX1/4 (E), GTX2/3 (F), dcGTX2/3 (G), GTX5 (H), GTX6 (I), C1/2 (J) and Mix1 and Mix2 (K) reference after periodate oxidation and vanadium pentoxide oxidation. Mix1 is a mixture of STX, dcSTX, GTX1/4, GTX5 and dcGTX2/3. Mix2 is a mixture of STX, neoSTX, dcSTX, dcneoSTX, GTX1/4, GTX2/3, GTX5, GTX6, dcGTX2/3 and C1/2. The peak area of main oxidation products is shown besides each chromatographic peak.

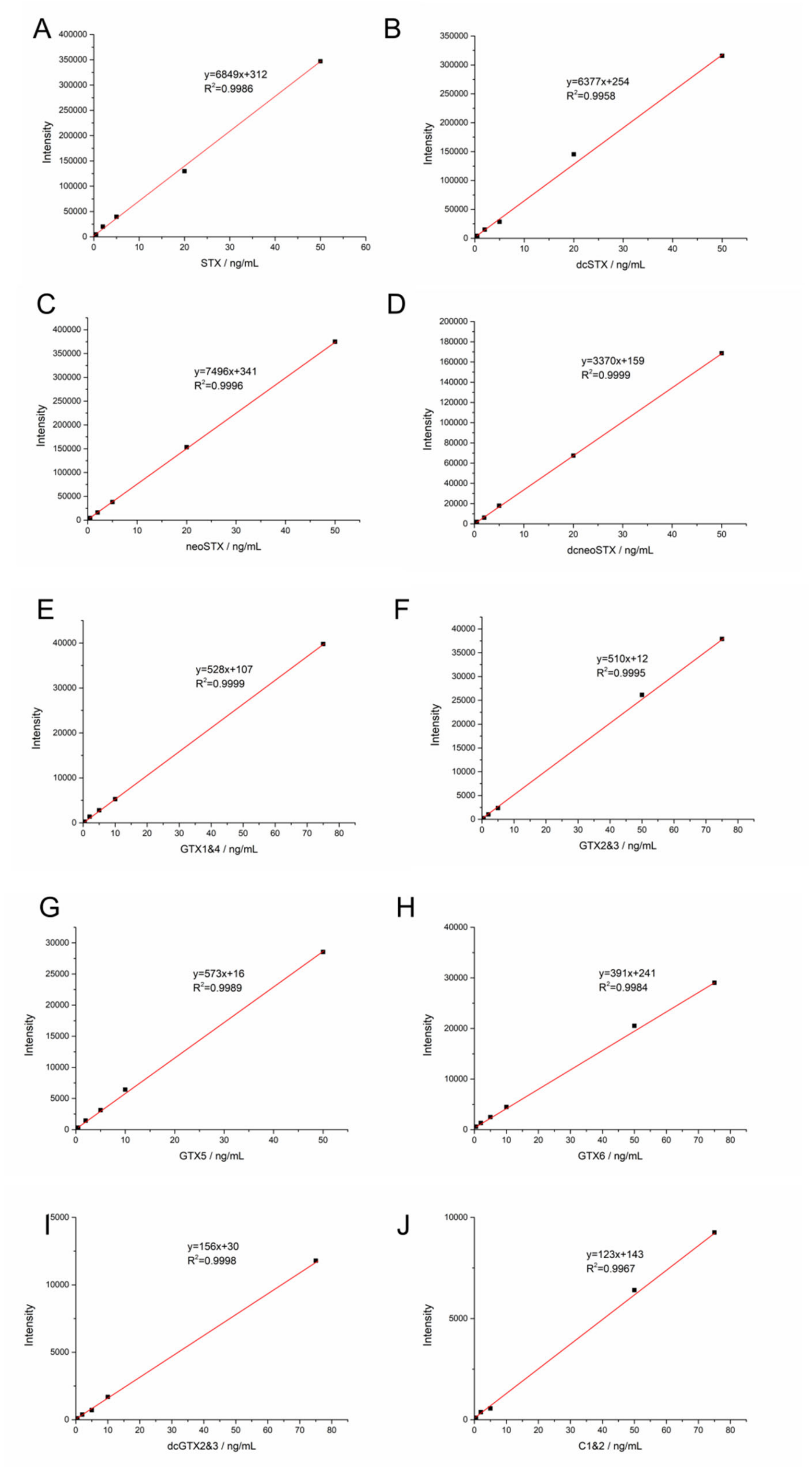

Figure S5. Calibration plots of STX (A), dcSTX (B), neoSTX (C), dcneoSTX (D), GTX1/4 (E), GTX2/3 (F), GTX5(G), GTX6 (H), dcGTX2/3 (I), and C1/2 (J) concentration against mass spectrum intensity for their main oxidation products after vanadium pentoxide oxidation.

Table S1. Optimized ion transition parameters for PSTs oxidation products.

**Target compound**	**Precursor ion *m/z***	**Quantitative ion *m/z***	**Collision energy (eV)**	**Qualitative ion *m/z***	**Collision energy (eV)**	**Detection mode**
**P1**	253	235	15	151	25	+
**P2**	296	235	14	151	17	+
P3	269	151	35	251	15	+
**P4**	312	251	15	209	25	+
	390	310	14	179	25	-
**P5**	269	163	28	209	24	+
	347	179	30	267	17	-
**P6**	376	296	7	235	20	+
	374	302	15	122	20	-
**P7**	392	312	7	251	20	+
	470	390	15	347	20	-

Table S2. Reaction products after oxidation with vanadium pentoxide.

PSTs	Product 1	Percentage of peak/%	Product 2	Percentage of peak/%
STX	P2	100		
dcSTX	P1	100		
neoSTX	P2	99.3	P1	0.7
dcneoSTX	P1	99.1	P3	0.9
GTX1/4	P4	99.4	P5	0.6
GTX2/3	P4	99.4	P5	0.6
GTX5	P6	65.5	P1	34.5
GTX6	P6	65.4	P1	34.6
dcGTX2/3	P5	100		
C1/2	P5	49.3	P7	50.7

Table S3. Stability of main PSTs oxidation products after vanadium pentoxide oxidation.

Main oxidation products of	Analyte	Concentration (ng/mL)	Stability (%)	Concentration (ng/mL)	Stability (%)
STX	P2	1.0	93.1	50	93.4
dcSTX	P1	1.0	94.1	50	93.3
neoSTX	P2	1.0	92.3	50	91.3
dcneoSTX	P1	1.0	97.2	50	93.6
GTX1/4	P4	3.0	89.7	50	84.7
GTX2/3	P4	3.0	87.4	50	85.8
GTX5	P6	1.5	92.3	50	92.0
GTX6	P6	1.5	92.9	50	89.8
dcGTX2/3	P5	3.0	91.0	50	92.2
C1/2	P7	5.0	87.1	50	89.9

Table S4. Comparison of limits of detection for PSTs in plasma between the present method and HILIC-MS/MS approach.[39]

Toxin	This work		HILIC-MS/MS	
	**LOD/(μg/L)**	**LOQ/(μg/L)**	**LOD/(μg/L)**	**LOQ/(μg/L)**
STX	0.3	1.0	1.4	4.2
dcSTX	0.3	1.0	0.61	1.82
neoSTX	0.3	1.0	0.58	1.75
dcneoSTX	0.3	1.0	1.14	3.43
GTX1/4	1.0	3.0	0.68	2.03
GTX2/3	1.0	3.0	0.98	2.94
GTX5	0.5	1.5	2.4	7.21
GTX6	0.5	1.5	0.56	1.68
dcGTX2/3	1.0	3.0	4.01	12.04
C1/2	1.5	5.0	1.54	4.62
